# EFTUD2 is a promising diagnostic and prognostic indicator involved in the tumor immune microenvironment and glycolysis of lung adenocarcinoma

**DOI:** 10.3389/fonc.2025.1499217

**Published:** 2025-04-01

**Authors:** Ankang Yin, Yufan Xu, Xiyang Su, Runan Wang, Zebin Zhang, Yi Chen, Lu Han, Guoxiang Fu, Wei Wang, Juan Wang

**Affiliations:** ^1^ School of Medical Technology and Information Engineering, Zhejiang Chinese Medical University, Hangzhou, Zhejiang, China; ^2^ Department of Pathology, Sir Run Shaw Hospital, Zhejiang University School of Medicine, Hangzhou, Zhejiang, China; ^3^ Department of Laboratory Medicine, The Second Affiliated Hospital of Zhejiang Chinese Medical University, Hangzhou, Zhejiang, China; ^4^ Department of Pathology, Tongde Hospital of Zhejiang Province, Hangzhou, Zhejiang, China; ^5^ Department of Clinical Laboratory, Key Laboratory of Cancer Prevention and Therapy Combining Traditional Chinese and Western Medicine of Zhejiang Province, Zhejiang Academy of Traditional Chinese Medicine, Tongde Hospital of Zhejiang Province, Hangzhou, Zhejiang, China

**Keywords:** lung adenocarcinoma, EFTUD2, prognosis, immune infiltration, glycolysis

## Abstract

**Background:**

Elongation Factor Tu GTP Binding Domain Containing 2 (EFTUD2), a conserved spliceosomal GTPase, is involved in craniofacial development and various cancers, but its role in lung adenocarcinoma (LUAD) remains unclear.

**Methods:**

EFTUD2 expression in LUAD tissues was analyzed using data from TCGA and GEO, and validated by immunohistochemistry, RT-qPCR, and Western blotting. The relationship between EFTUD2 expression and clinical features was examined using Fisher’s exact test. Diagnostic and prognostic analyses were performed in R. Hub genes related to EFTUD2 were identified through topological algorithms, and immune infiltration was assessed using CIBERSORT. The cGAS-STING pathway and m6A modification were also analyzed in the TCGA LUAD cohort. Functional assays were conducted to assess EFTUD2’s impact on LUAD cell proliferation, cell cycle, invasion, and metastasis, while glycolytic enzyme levels were measured by Western blotting.

**Results:**

EFTUD2 was upregulated in LUAD tissues and cells, correlating with N classification, visceral pleural invasion, intravascular tumor embolism, and cytokeratin-19 fragment antigen 21-1. Sixteen EFTUD2-related hub genes were identified. Higher EFTUD2 expression was linked to altered immune cell infiltration, with increased TumorPurity scores and decreased StromalScore, ImmuneScore, and ESTIMATEScore values. Gene enrichment analyses highlighted EFTUD2’s involvement in cell adhesion, immune response. EFTUD2 was strongly associated with the cGAS-STING pathway and m6A modification. EFTUD2 knockdown inhibited LUAD cell proliferation, migration, and tumorigenicity, causing G0/G1 phase cell cycle arrest, and altered glycolytic enzyme expression. These findings may suggest that EFTUD2 positively regulates the progression of LUAD and modulates the glycolytic activity of tumor cells, making it valuable for LUAD treatment and prognosis.

**Conclusions:**

EFTUD2 is a potential diagnostic and prognostic marker for LUAD, associated with immune infiltration, the tumor microenvironment, the cGAS-STING pathway, m6A modification, and glycolysis.

## 1 Introduction

Lung cancer is a major contributor to cancer-related death globally, and lung adenocarcinoma (LUAD) represents approximately half of all reported lung cancer cases (1, 2). According to Siegel et al., as of 2024, lung cancer remains the leading cause of cancer-related deaths. The mortality rates for male and female lung cancer patients are 20% and 21%, respectively. The incidence of lung cancer is 49.0 cases per 100,000 individuals annually. Among newly diagnosed cancer cases, lung cancer accounts for 11% in men and 12% in women (2). Because LUAD presents with obscure clinical symptoms, the majority of the affected patients are diagnosed at an advanced stage, typically after the optimal window for surgical removal, and thus have a 5-year survival rate of ≤20%. Nevertheless, early detection and intervention can increase the LUAD survival rate to 60% (2, 3). Common cancer therapies include surgery, radiation therapy, chemotherapy, and allogeneic hematopoietic stem cell transplantation. In the future, treatments targeting genes with oncogenic potential will become one of the key approaches in cancer therapy (4). Low-dose computed tomography (LDCT) that can be used to identify early-stage tumors has facilitated the reduction of mortality rates among high-risk LUAD patients (3). However, LDCT, which can also detect benign nodules, is associated with a 25% false positive rate for nodule detection; this reduces the positive predictive value of LDCT and narrows its therapeutic applicability to larger groups (3). Hence, discovering biomarkers for selecting patients eligible for LDCT assessment is highly warranted. In recent years, significant progress has been made in the discovery and application of emerging biomarkers. Emerging biomarkers for LUAD include long non-coding RNAs (lncRNAs), microRNAs (miRNAs), epigenetic markers, as well as coding RNAs and protein markers (5–7).

Elongation factor Tu GTP binding domain containing 2 (EFTUD2), a highly conserved spliceosomal GTPase and alternative splicing factor, is a key constituent of U5 small nuclear ribonucleoproteins; as such, EFTUD2 is essential for spliceosome activation and RNA-splicing process regulation (8, 9). The maintenance of spliceosome dynamics depends on the binding of GTP/GDP to EFTUD2; however, disruption of this binding can prevent mRNA splicing, potentially leading to cell death (10, 11). Mutations or insufficient expression of EFTUD2 can result in abnormalities in craniofacial development (12–16). EFTUD2 also acts as an innate immunity regulator (17), playing a crucial role in hepatitis C and B virus infection prevention (18, 19).

Several recent studies have highlighted the role of EFTUD2 in cancer progression. EFTUD2 has been noted to increase hepatocellular carcinoma (HCC) cell survival and metastasis by inducing epithelial–mesenchymal transition and be associated with immune infiltration and poor prognosis, making it a potential therapeutic target for liver cancer (20–22). In their clinical study, Beyer et al. noted that increased EFTUD2 expression in endometrial cancer predicts poor prognosis and thus can be an independent indicator of progression-free survival (23). EFTUD2 is strongly expressed in colorectal cancer cells, and its deficiency in myeloid progenitor cells can inhibit cancer occurrence and progression (24). In general, EFTUD2 is strongly associated with cancer progression; however, the function of EFTUD2 in LUAD remains unknown.

In the present study, we first determined that EFTUD2 is strongly expressed in LUAD by using data from online databases. We then confirmed that EFTUD2 is highly expressed in LUAD tissue samples, indicating its potential as a LUAD diagnostic marker and prognostic indicator. Next, the hub genes associated with EFTUD2 were screened on the basis of The Cancer Genome Atlas (TCGA) data. Our results demonstrated that EFTUD2 shaped the tumor immune microenvironment (TIME) and is closely related to genes involved in N6-methyladenosine (m6A) modification and the cyclic GMP-AMP synthase-stimulator of interferon genes (cGAS-STING) pathway. Moreover, EFTUD2 knockdown was noted to inhibit the proliferation, migration, invasion, tumorigenicity, and cell cycle of LUAD cells. Gene set enrichment analysis (GSEA) revealed the signaling pathways in which EFTUD2 in LUAD cells might be involved. In general, by leveraging bioinformatics analysis followed by experimental substantiation, we elucidated the prospective function and predictive value of EFTUD2 in LUAD, indicating that EFTUD2 is a promising candidate for a therapeutic target in LUAD.

## 2 Methods

### 2.1 Clinical samples

We obtained 174 lung clinical samples surgically excised and pathologically confirmed to be of LUAD from Sir Run Run Shaw Hospital, Affiliated with Zhejiang University, China, over 2018–2021. The 174 clinical surgically resected tissue samples include both tumor and paracancerous regions. These samples were from retrospectively enrolled patients who had not received any radiotherapy or chemotherapy presurgically. All patients were adults aged 34–86 years. We determined the clinical characteristics for analysis based on the research direction and the patient information available, which include Sex, Age, Smoking history, Visceral pleural invasion, Intravascular tumor embolism, TNM stages, and levels of LUAD-related biomarkers (CA125, CEA, CYFRA-21-1, Pro-GRP). To ensure the completeness of the analysis, we selected patients who met all the clinical characteristics mentioned above, patients who were missing any of the criteria will be excluded. This process was entirely random and involved no subjectivity. All experiments on the clinical samples were approved by the Research Ethics Committee of Sir Run Run Shaw Hospital, Affiliated with Zhejiang University.

### 2.2 Hematoxylin–eosin staining

The 174 lung clinical samples obtained in section 2.1 are paraffin-embedded sections of surgically resected LUAD tissues. The sections were sequentially placed in xylene I for 8 min, xylene II for 8 min, xylene III for 8 min, absolute ethanol I for 5 min, absolute ethanol II for 5 min, 85% ethanol for 5 min, 75% ethanol for 5 min, and washed with water for 2 min. The sections were stained with hematoxylin for 5 min, differentiated in hydrochloric acid solution for 2 s, then blued in ammonia water solution for 15–30 s, followed by rinsing with water. Afterward, the sections were immersed in 95% ethanol for dehydration, then stained in eosin solution for 5–8 s. The sections were sequentially placed in absolute ethanol I for 30 s, absolute ethanol II for 2.5 min, absolute ethanol III for 2.5 min, xylene I for 2.5 min, xylene II for 2.5 min, and finally mounted with neutral gum. The sections were examined under a microscope, and images were captured for analysis.

### 2.3 Immunohistochemical staining

The paraffin-embedded sections were deparaffinized, rehydrated, and subjected to antigen retrieval. The tissue sections were placed in a retrieval box filled with sodium citrate buffer (pH 6.0). Once the buffer inside the pressure cooker reached boiling, the retrieval box was carefully placed inside. Antigen retrieval was performed at 121°C under high pressure for 3 minutes. Afterward, the pressure was released, and the sections were allowed to cool naturally to room temperature. The slides were then transferred to PBS and washed on a decolorizing shaker three times for 5 minutes each. Next, they were incubated in 3% hydrogen peroxide at room temperature for 25 min, followed by washing with 1× phosphate-buffered saline (PBS). The tissue sections were evenly covered with 3% bovine serum albumin and incubated at room temperature for 30 min. The sections were then incubated with the rabbit EFTUD2 polyclonal antibody (Abcam Cat# ab72456, RRID: AB_1268731) at 4°C overnight. The dilution of the EFTUD2 antibody for IHC staining is 1:200. After three PBS washes, the sections were incubated with a goat antirabbit secondary antibody conjugated with poly-horseradish peroxidase at room temperature for 50 min and visualized using a 3,3’-diaminobenzidine chromogen substrate. The sections were counterstained with hematoxylin for 3 min, dehydrated, air-dried, and finally, sealed with a neutral resin. Two independent pathologists (GX Fu and YF Xu) specializing in LUAD evaluated all sections.

EFTUD2 expression was calculated according to the tumor cell staining intensity and staining positivity rate. Staining intensities were rated as follows: 0, negative staining; 1, weak staining; 2, moderate staining; and 3, strong staining. Subsequently, the final IHC score for each sample was obtained by summing the range of positive cells and the staining intensity of the EFTUD2 protein. Moreover, positivity rates were scored from 0 to 4 as follows: 0, 0%; 1, 1%–25%; 2, 26%–50%; 3, 51%–75%; and 4, >75%. The final scores were a sum of the two aforementioned scores and categorized as follows: negative, <3; weak, 3; moderate, 4; and strong, ≥5 (25).

### 2.4 EFTUD2 expression in LUAD datasets

The GSE32863 (26), GSE43458 (27), and GSE75037 (28) datasets from the Gene Expression Omnibus (GEO) database (29) were downloaded to analyze EFTUD2 expression in LUAD and normal samples. The criteria for selecting the GSE32863, GSE43458, and GSE75037 datasets is as follows: (1) LUAD tissue derived from human species; (2) Like the TCGA dataset, LUAD patients are from the USA; (3) The number of cancer samples is greater than 50, and the combined number of cancer and adjacent normal samples is greater than 100. EFTUD2 expression was also assessed in TCGA datasets (30) by using the web-based tool Gene Expression Profiling Interactive Analysis (GEPIA; version 2.0). Clinical factors, including age, sex, and pathologic (tumor: T, node: N, and metastasis: M) stages et al., were obtained from TCGA and used to group the samples. In the TNM staging system, T represents the size of the tumor and the extent of the primary tumor, N indicates the involvement of regional lymph nodes, and M refers to the presence of distant metastasis. The Kruskal–Wallis test was used to compare EFTUD2 expression among different clinical factor groups in R (version 4.1.2; RRID: SCR_001905). The diagnostic and prognostic value of EFTUD2 in patients with LUAD was assessed using receiver operating characteristic (ROC) curve and Kaplan–Meier (K–M) survival analyses. The mutation MAF data for LUAD samples were downloaded from the Genomic Data Commons (GDC, version 2.15.5). The Tumor Mutation Burden (TMB) and Fraction of Genome Altered (FGA) for these samples were then calculated using maftools (version 2.6.05) in R (version 4.3.1). Subsequently, the cor function (version 4.1.2) in R was used to calculate the correlations between TMB, FGA, and the expression levels of EFTUD2.

### 2.5 Analysis of correlation between EFTUD2 and clinical characteristics

Based on the median staining scores, immunohistochemical (IHC) staining results of the all patient clinical samples were categorized into high and low EFTUD2 expression groups (n = 91 and 83, respectively). Subsequently, two clinical characteristic data categorical variables were obtained, using the high and low expression levels of EFTUD2 as the classification criteria. The correlation between clinical characteristics and EFTUD2 expression was analyzed by the univariate chi-square test. The clinical characteristics of the study include: Sex, Age, Smoking history, Visceral pleural invasion, Intravascular tumor embolism, TNM stages, and levels of LUAD-related biomarkers (CA125: carbohydrate antigen 125; CEA: carcinoembryonic antigen; CYFRA-21-1: cytokeratin-19 fragment antigen 21-1; Pro-GRP: pro-gastrin releasing peptide precursor). Moreover, and TCGA samples were divided into high- and low-expression groups based on their median EFTUD2 expression. Then, we used Fisher’s exact test to compare each clinical factor’s distribution between the two groups in R (version 4.1.2).

### 2.6 Differentially expressed gene screening

TCGA gene expression profiles based on the Illumina HiSeq 2000 platform were downloaded from the UCSC-Xena database (31), which yielded 559 samples, including 501 LUAD tumor and 58 normal control samples. Differential expression analysis was then performed on the LUAD and control groups using the R package limma (version 3.56.2). Genes with a false discovery rate (FDR) of <0.05 and |log_2_ fold change (FC)| of >1 were considered significantly differentially expressed.

### 2.7 Gene set enrichment analysis

GSEA of LUAD genes was performed using GSEA (version 4.3.2) (32) based on the median EFTUD2 expression, with the screening thresholds set to FDR (q) < 0.05 and *p* < 0.001.

### 2.8 Protein–protein interaction network creation and EFTUD2-related hub gene identification

We constructed a protein–protein interaction (PPI) network of EFTUD2 with the screened differentially expressed genes (DEGs), cGAS-STING pathway–related genes, and m6A modification–related genes by using the STRING database (version 12.0) (33). The network was visualized using Cytoscape (version 3.9.0). This EFTUD2 PPI was then analyzed for hub genes by using the cytoHubba plugin (version 0.1) of Cytoscape (3.9.0), based on these 12 topological analysis algorithms: maximal clique centrality, degree-based maximal neighborhood centrality, maximal neighborhood centrality, edge percolated centrality, Degree, BottleNeck, Closeness, Eccentricity, Betweenness, Radiality, Stress, and Clustering Coefficient. The genes commonly selected by at least nine of the algorithms were retained as the final hub genes. The comparison results were displayed using the R package UpSetR (version 1.4.0).

### 2.9 Correlation analysis

We performed correlation analysis to evaluate the relationships of EFTUD2 with the hub genes, immune cells, ESTIMATE scores, immune-related genes, cGAS-STING pathway–related genes, and m6A modification–related genes using the cor function in R. The Gene-Corr module of TIMER (version 2.0) (34) was used to assess correlations between EFTUD2 and glycolytic genes in LUAD.

### 2.10 Diagnostic and prognostic analysis

ROC analysis (35) and Cox analysis (36) are widely used and recognized as common single-gene independent diagnostic methods, making them highly suitable for assessing the diagnostic and prognostic value of EFTUD2 in LUAD. The diagnostic value of EFTUD2 and the hub genes was assessed using ROC curve analysis in R package pROC (version 1.12.1). The survival of *EFTUD2*, immune cells, immune scores, immune genes, cGAS-STING pathway–related genes, and m6A modification–related genes was analyzed using the R package survival (version 2.41-1). Based on the clinical information from the TCGA dataset samples and the median grouping of EFTUD2 gene expression levels, Cox univariate and multivariate regression analyses were performed using the survival package (Version 2.41-1) in R (version 4.1.2).

### 2.11 Immune infiltration analysis

Immune cell proportions in the TCGA tumor samples were assessed using CIBERSORT (RRID: SCR_016955) (37), followed by the calculation of ESTIMATE scores, immune scores, stromal scores, and tumor purity by using the R package estimate (version 1.0.13). Finally, the distributions of immune cells, ESTIMATE scores, immune scores, stromal scores, and tumor purity were compared between the high- and low-expression groups.

### 2.12 Identification of EFTUD2-related immune genes and gene annotation

Immune scores with differential EFTUD2 distribution were obtained on the basis of EFTUD2 expression levels. Subsequently, the correlation between these differential associated immune scores and the abovementioned DEG was assessed using the cor function in R (version 4.1.2). All selected immune-related genes associated with EFTUD2 had *p* < 0.05 and |cor| > 0.3. These immune-related genes were then subjected to Gene Ontology (GO) and Kyoto Encyclopedia of Genes and Genomes (KEGG) enrichment analysis based on DAVID (version 6.86) (38, 39), with FDR < 0.05 as the selection threshold.

### 2.13 Selection of DEGs associated with m6A modification and the cGAS-STING pathway

Expression data for genes related to m6A modification and the cGAS-STING pathway were extracted from the TCGA LUAD expression profile. The included genes were related to methylation (*METTL3*, *METTL14*, *METTL15*, *WTAP*, *VIRMA*, *RBM15*, *RBM15B*, and *ZC3H13*), demethylation (*FTO* and *ALKBH5*), m6A regulation (*RBMX*, *YTHDC1*, *YTHDC2*, *IGF2BP1*, *IGF2BP2*, *IGF2BP3*, *YTHDF1*, *YTHDF2*, *YTHDF3*, *HNRNPA2B1*, and *HNRNPC*), and the cGAS-STING pathway (*SAMHD1*, *DDX41*, *IRF3*, *PRKDC*, *XRCC5*, *XRCC6*, *TRIM21*, *TBK1*, *DTX4*, *STAT6*, *IFI16*, and *NLRC3*). The limma package (version 3.56.2) was used to compare the gene expression levels between the LUAD and normal control groups. To assess whether genes associated with EFTUD2 expression are influenced by their own copy number variations, mutation analysis, including CNV and SNV, was performed on the EFTUD2-related differentially expressed genes in the TCGA-LUAD dataset using the cBioPortal online platform (version 6.0.23).

### 2.14 Cell culture and transfection

MRC-5 (RRID: CVCL_0440), A549 (RRID: CVCL_0023), H1299 (RRID: CVCL_0060), and PC9 (RRID: CVCL_B260) cell lines (all from American Type Culture Collection) were cultured in Dulbecco’s modified Eagle medium (C11995500BT; Gibco, USA) supplemented with 10% fetal bovine serum (FBS; 164210; Pricella, China) and penicillin–streptomycin (BL505A; Labgic, China) at 37°C in a humidified incubator containing 5% CO_2_ and 95% air. Small interfering RNA (siRNA) targeting *EFTUD2* (GenePharma, China) and Lipofectamine 2000 (11668019; Invitrogen, USA) were separately diluted in OPTI-MEM (31985070; Gibco, USA), followed by incubation for 5 min. Then, the two solutions were mixed, followed by incubation for an additional 15 min. This mixture was added to the cells in a six-well plate, followed by incubation for 6–8 h. The final concentration of siRNA used for transfection was 100 nM. Finally, the transfection medium was replaced with the regular culture medium. Subsequently, the transfected cells were cultured in a 37°C incubator with 5% CO2 for 48 h. All *EFTUD2*-targeting siRNA sequences are listed in the **Supplementary Table S2**.

### 2.15 Total RNA extraction and reverse transcription real-time polymerase chain reaction

Total RNA was extracted using a TRIzol reagent kit (9109; Takara Bio, Japan). The extracted RNA was then reverse-transcribed into cDNA using a reverse transcription kit (RR047A; Takara Bio, Japan). Reverse transcription real-time polymerase chain reaction (qRT-PCR) was then performed using TB Green qPCR mix (RR420; Takara Bio, Japan), according to the manufacturer’s instructions. All primers used here, synthesized by Tsingke (China), are presented in the **Supplementary Table S1**. The qRT-PCR results were analyzed using the 2^−ΔΔCT^ method.

### 2.16 Western blotting

Prepared cells were lysed in a cell lysis buffer (Cell Signaling Technology, Danvers, USA) at 4°C. Protein quantification was performed using a bicinchoninic acid protein assay kit (P1511; Applygen, China). Western blotting was performed in a sodium dodecyl sulfate–polyacrylamide gel electrophoresis system. The following primary antibodies were used: rabbit EFTUD2 polyclonal antibody (Abcam Cat# ab72456, RRID: AB_1268731, dilution factor: 1: 2000), rabbit anti-GAPDH polyclonal antibody (Cell Signaling Technology Cat# 2118, RRID: AB_561053, dilution factor: 1: 1000), rabbit anti-PKM2-specific antibody (Proteintech Cat# 15822-1-AP, RRID: AB_1851537, dilution factor: 1: 2000), rabbit anti-ALDOB polyclonal antibody (Cohesion Biosciences Cat# CPA3123, RRID: AB_3106870, dilution factor: 1: 1000), and rabbit anti-β-actin polyclonal antibody (Cell Signaling Technology Cat# 4970, RRID: AB_2223172, dilution factor: 1: 1000). TBST (Tris Buffered Saline with Tween-20) supplemented with 5% fetal bovine serum (FBS) was used as the antibody diluent. The dilution of the antibodies was determined according to the manufacturer’s instructions.

### 2.17 Flow cytometric cell-cycle analysis

Flow cytometry was used to analyze the cell cycle (40). Cells were prepared and digested with 0.25% trypsin without ethylenediaminetetraacetic acid to obtain single-cell suspensions at concentrations of 1 × 10^5^ to 5 × 10^5^ cells/mL. The cells were then washed twice with PBS, and the supernatant was discarded. Cell-cycle analysis was conducted using a Cell Cycle kit (C543; Dojindo, Japan), according to the manufacturer’s instructions. The flow cytometry results for the cell cycle were analyzed and plotted using Flowjo (version 7.6).

### 2.18 Plate clone assay

Prepared single cells were seeded into a six-well plate at a density of 1,000 cells per well and cultured for 10–12 days. Cell culture was terminated when visible clones appeared, and the supernatant was discarded. The cells were then gently washed twice with PBS and fixed in 4% paraformaldehyde for 15 min, followed by three PBS washes. Subsequently, the cells were exposed to an appropriate amount of 0.1% crystal violet for 3 min. The plates were photographed, and clone numbers were calculated using ImageJ (version 1.54h).

### 2.19 CCK-8 assay

The treated cell suspensions were seeded at a density of 5,000 cells in 100 μL per well in a 96-well plate. The CCK-8 solution (CCK-8; C543; Dojindo, Japan) was mixed with a serum-free medium at a 1:10 ratio and used to replace the cell culture medium, followed by incubation at 37°C for 1–4 h. The optical density was measured at 450 nm on an MRX II microplate reader (Dynex, USA). Each experiment was performed independently in three replicates.

### 2.20 Transwell assay

Matrigel (1567ML005; Neofroxx, Germany) was first diluted and then added to the upper chamber of a transwell plate. Next, a single-cell suspension was added to the upper chamber of the transwell plate, whereas a medium containing 10% FBS was added to the lower chamber. This was followed by incubation at 37°C for 48 h, fixing with 4% paraformaldehyde for 30 min, and finally, staining with 0.1% crystal violet for 3 min. Cells that did not pass through the Matrigel in the upper chamber were gently wiped away with a wet cotton swab. Images were captured under a fluorescence microscope (Olympus, Tokyo, Japan).

### 2.21 Wound healing assay

Cells were seeded at a density of approximately 3 × 10^5^ cells per well in a 6-well plate, with 2 mL of DMEM medium containing 5% FBS added to each well. After incubation for 12 h in a 37°C incubator with 5% CO2, scratches were made in each well using a 10 μL pipette tip along a sterile ruler. The detached cells were removed by washing with sterile 1 × PBS, and 2 mL of serum-free medium was added to each well. The plates were then placed back in a 37°C incubator with 5% CO2. Microscopic images were taken at the same location at 0 and 48 h. The scratch width was measured using ImageJ (version 1.54h).

### 2.22 Establishment of LUAD xenografts in nude mice

For the tumor xenograft experiments, 4-week-old male Balb/c nude mice weighing 18 ± 2 g were selected. The mice were housed in a specific pathogen-free (SPF) sterile environment, maintained at a constant temperature of 25°C with a 12-h light-dark cycle, and provided with ample food and water. The nude mice were randomly divided into two groups: a control group (si-NC) and an EFTUD2 knockdown group (si-EFTUD2), with 5 mice in each group, totaling 10 mice. A549 cells were used for modeling in the animal experiments, and the cell culture conditions were the same as described in section 2.14. A549 cells were digested with trypsin, collected by centrifugation at 1000 rpm for 5 min, and resuspended in PBS. The cells were washed twice by centrifugation and resuspension. Cell counting was performed, and the cell density was adjusted to 1×10^6^ cells/mL. The cell suspension was divided into 100 μL portions, and each portion was injected into the right axilla of the forelimb of each mouse. After cell inoculation, the mice were returned to normal housing. Once the tumor xenograft masses became palpable, the diameter of the tumors was recorded every 3 days, and growth curves for body weight and tumor size were plotted.

When the tumor xenograft diameter reached 5-7 mm, the knockdown group mice were treated with a mixture of 15 µg EFTUD2 siRNA and 5 μL Lipofectamine 2000 (11668019; Invitrogen, USA) every 3 days via an intratumoral multi-point injection method. The EFTUD2 siRNA used in the animal experiments was modified with 3’Cholesterol and 2’OMe, and the nucleotide sequence was the same as shown in **Supplementary Table S2**. The control group was injected with negative siRNA and 5 µL Lipofectamine 2000, with all other treatment conditions being the same as those for the knockdown group. Before euthanasia, blood samples were taken from the mice. At the end of the experiment, the mice were euthanized by cervical dislocation, and their appearance was photographed for records. Tumor xenografts were then excised, photographed, measured, and recorded.

### 2.23 Measurement of lactate levels in mouse serum and tumor xenografts

The Lactate Assay Kit (BC2235; Solarbio, China) was used to measure lactate levels in serum and tumor xenografts. The specific steps were performed according to the protocol provided with the kit.

### 2.24 Pre-treatment for IHC and HE staining of mouse tumor xenografts

The mouse tumor xenografts were placed into dehydration cassettes and dehydrated using a dehydration machine (Donatello; DIAPATH, China) according to the following program: 75% ethanol for 4 h, 85% ethanol for 2 h, 90% ethanol for 2 h, 95% ethanol for 1 h, anhydrous ethanol I for 30 min, anhydrous ethanol II for 30 min, ethanol-xylene for 5 min, xylene I for 5 min, xylene II for 5 min, melted paraffin I at 65°C for 1 h, melted paraffin II at 65°C for 1 h, and melted paraffin III at 65°C for 1 h. The tumor xenografts were then placed in embedding molds with the melted wax, and before the wax solidified, the embedding molds were placed on a -20°C cold plate to cool. After the wax solidified, the wax blocks were removed from the molds and sectioned. The IHC and HE staining procedures for the mouse tumor xenograft specimens are described in sections 2.2 and 2.3. The IHC results from the animal experiments are expressed as the percentage of positive cells. The antibodies used for IHC staining of the mouse tumor xenografts, including EFTUD2, GAPDH, PKM2, and ALDOB, are the same as those used in the section 2.16.

### 2.25 TUNEL assay

On sections of the mouse tumor xenograft, 100 µL of proteinase K solution (E00492; Fermentas, Canada) at a concentration of 20 µg/mL was added and incubated at room temperature for 20 min. Afterward, the sections were washed with PBS for 3 min, repeating this step 3 times. The treated samples were placed in a humidified box to maintain moisture. Then, 100 µL of 3% H_2_O_2_ solution was applied to completely cover each sample and incubated at room temperature for 5 min. The samples were then rinsed with PBS and air-dried. The TUNEL reaction mixture (G1507; Servicebio, China) was prepared, and TUNEL staining was performed according to the instructions provided with the kit. After completing the TUNEL staining, the samples were counterstained with hematoxylin. The samples were immersed in hematoxylin (G1004; Servicebio, China) for 5 min, rinsed with distilled water, treated with hematoxylin differentiator (from the hematoxylin kit) for 2 s, rinsed again with distilled water, and then treated with hematoxylin bluing solution (from the hematoxylin kit) for 5 s, followed by another rinse with distilled water. The samples were immersed in absolute ethanol for dehydration 4 times, each for 5 min; immersed in xylene for 5 min, then transferred to fresh xylene for another 5 min. Finally, the slices were sealed with neutral gum. The samples were examined under a brightfield microscope. The nuclei of apoptotic cells appeared brownish-yellow, while the nuclei of normal cells appeared blue.

### 2.26 Statistical analysis

Public data from the TCGA and GEO databases were statistically processed and analyzed using R (version 4.1.2). Graphs and charts were created using both GraphPad Prism (version 9.0) and R (version 4.1.2). The overall survival (OS) was analyzed using the log-rank test. Relationships between two continuous variables were assessed using Pearson or Spearman analysis. Moreover, between-group comparisons were performed using Student’s *t* test or one-way analysis of variance, and categorical data were analyzed using the chi-square test. Cox univariate and multivariate analyses were used to investigate the independent prognostic value of EFTUD2 in LUAD. ImageJ (version 1.54h) was used for quantitative analysis of protein band intensities in Western blotting. All quantitative data are expressed as means ± standard deviations (SDs). Each experiment was independently performed at least three times to ensure reproducibility. Significant differences were defined based on a *p* value or FDR of <0.05.

## 3 Results

### 3.1 EFTUD2 expression is upregulated in patients with LUAD

**Figure 1:**
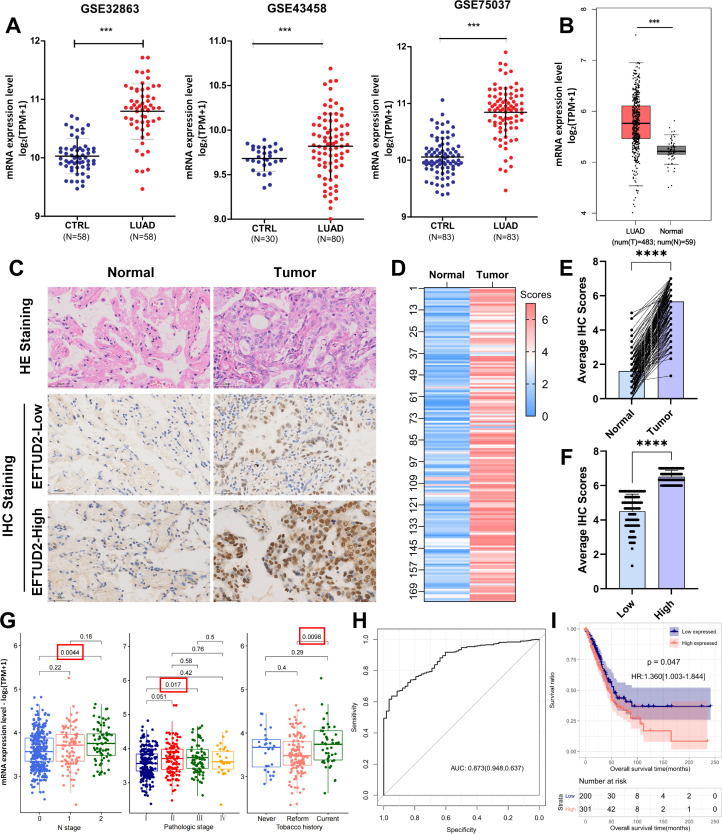
Significant prognostic and diagnostic value of elevated EFTUD2 expression in LUAD. **(A)** Comparative analysis of EFTUD2 expression in normal and LUAD tissues across the GEO datasets GSE32863 (normal: n=58; tumor: n=28), GSE43458 (normal: n=30; tumor: n=80), and GSE75037 (normal: n=83; tumor: n=83). FDR < 0.05. **(B)** EFTUD2 expression in normal lung and LUAD tissues from TCGA dataset (normal: n=59; tumor: n=483). **(C)** HE and IHC staining of LUAD and adjacent normal tissue sections. **(D)** Heatmap of EFTUD2 IHC scores in LUAD and adjacent normal tissue sections. **(E)** Histogram of EFTUD2 expression levels in normal and tumor tissues. **(F)** High and low EFTUD2 expression groups divided based on median IHC scores. **(G)** EFTUD2 expression across clinical factors (TNM stage, pathology, and smoking history) analyzed using Kruskal–Wallis test **(H)** ROC curve analysis for EFTUD2 diagnosis. **(I)** OS analysis of low (blue) and high (red) EFTUD2 expression groups. ***p < 0.001, ****p < 0.0001.

To determine *EFTUD2* mRNA expression profiles, we analyzed the GEO datasets GSE32863, GSE43458, and GSE75037. Compared with the normal tissues in GEO datasets, the LUAD tissues demonstrated significantly higher *EFTUD2* expression (GSE32863: *p* < 0.001, GSE43458: *p* < 0.001, and GSE75037: *p* < 001; **Figure 1A**). Elevated EFTUD2 expression was also verified in TCGA LUAD dataset (**Figure 1B**). Among clinical samples, the HE staining results demonstrated that LUAD tumor cells exhibited an irregular glandular invasive growth pattern, with weakly eosinophilic or clear cytoplasm (**Figure 1C**). Their nuclei were enlarged and irregular with uneven chromatin distribution and had visible mitotic figures (**Figure 1C**). Their stroma was accompanied by a desmoplastic fibrotic tissue reaction and showed chronic inflammatory cell infiltration (**Figure 1C**). Next, EFTUD2 expression in 174 paraffin-embedded primary LUAD tissues was assessed through IHC staining. The heatmap in **Figure 1D** illustrates differences in EFTUD2 expression between LUAD tissues and adjacent normal tissues in every sample: EFTUD2 expression was significantly higher in the LUAD tissues than in the adjacent tissues (**Figure 1E**), and the high expression group demonstrated significantly higher median EFTUD2 expression than the low-expression group (**Figure 1F**). The distribution of EFTUD2 expression in LUAD patients harboring EFTUD2 mutations was assessed. The findings revealed a very low frequency of EFTUD2 SNVs in LUAD patients (n = 4), and no significant differences in EFTUD2 expression were observed between the missense and non-mutated groups (**Supplementary Figure S1A**). In contrast, EFTUD2 mRNA expression was more markedly influenced by CNVs, with significantly elevated expression levels in the Gain and Amplification groups compared to the diploid group, and the Amplification group exhibiting the most pronounced increase (**Supplementary Figure S1B**). While a few Shallow Deletion mutations were detected in LUAD patients, the number of affected individuals was minimal. Furthermore, EFTUD2 expression in LUAD demonstrated a significant positive correlation with the TMB (**Supplementary Figure S1C**) and FGA (**Supplementary Figure S1D**).

### 3.2 EFTUD2 is a LUAD biomarker, and its upregulation predicts poor prognosis

To explore whether high EFTUD2 expression is correlated with clinical characteristics and tumor biomarkers (20), we divided the collected patient samples into two groups according to median EFTUD2 expression and found that age (*p* = 0.0093), N classification (*p* = 0.0163), visceral pleural invasion (VPI; *p* = 0.0270), and intravascular tumor embolism (*p* = 0.0092) were significantly associated with EFTUD2 upregulation (**Table 1**). Moreover, EFTUD2 upregulation was also closely associated with cytokeratin-19 fragment antigen 21-1 (CYFRA-21-1), an independent prognostic factor in patients with LUAD (*p* = 0.0319; **Table 2**) (41). In addition to our clinical study, the TCGA results also demonstrated significant differences in EFTUD2 expression across the subgroups of pathologic N stage (N1 vs. N0), cancer stage (III vs. I), and smoking history (current smoker vs. previous smoker; **Figure 1G**). Significant distribution differences were noted in age, N stage, and smoking history between groups with high and low EFTUD2 expression (**Table 3**)—consistent with results presented in **Table 1**. The ROC curve for EFTUD2 from the TCGA-LUAD dataset demonstrated that EFTUD2 had an area under the ROC curve (AUC) of 0.873 (**Figure 1H**). The K–M survival analysis revealed that high EFTUD2 expression is associated with poor OS (**Figure 1I**). Cox analysis was performed for both univariate and multivariate analyses based on EFTUD2 expression levels and clinical characteristics to evaluate the independent prognostic value of EFTUD2 in LUAD. As shown in **Table 4**, the univariate analysis revealed that EFTUD2, TNM staging, and pathological staging exhibited good prognostic performance with HR > 1. However, the multivariate analysis results for EFTUD2 were not significant, suggesting that the prognostic value of EFTUD2 in LUAD may be related to the progression of the disease. Thus, EFTUD2 may be a LUAD biomarker, and its upregulation may predict poor prognosis.

**Table 1:** Relationship between EFTUD2 expression and clinical characteristics in patients with LUAD.

Characteristic	Group	Total	EFTUD2 expression		*P*
			Low (%)	High (%)	
All patients		174	92 (52.87)	86 (47.13)	
Sex					0.4361
	Male	102	53 (51.96)	49 (48.03)	
	Female	72	38 (52.78)	34 (47.22)	
**Age (years)**					**0.0093**
	≤ 65	108	64 (59.26)	44 (40.74)	
	> 65	66	27 (40.91)	39 (59.09)	
Smoking history					0.0757
	No	123	59 (47.97)	64 (52.03)	
	Yes	51	32 (62.75)	19 (37.25)	
**Visceral pleural invasion**					**0.0270**
	No	148	66 (44.59)	53 (55.41)	
	Yes	26	9 (34.62)	17 (65.38)	
	NA	29	16 (55.17)	13 (44.83)	
**Intravascular tumor embolism**					**0.0092**
	No	132	72 (54.55)	60 (45.45)	
	Yes	14	3 (21.43)	11 (78.57)	
	NA	28	16 (57.14)	12 (42.86)	
T stage					0.0769
	1	93	51 (54.84)	42 (45.16)	
	2	50	26 (52.00)	24 (48.00)	
	3	11	2 (18.18)	9 (81.82)	
	4	18	12 (66.67)	6 (33.33)	
	NA	2	0	2 (100.00)	
**N stage**					**0.0163**
	0	89	55 (61.80)	34 (38.20)	
	1	29	16 (55.17)	13 (44.83)	
	2	41	13 (31.71)	28 (68.29)	
	3	13	7 (53.85)	6 (46.15)	
	NA	2	0	2 (100.00)	
M stage					0.8419
	0	152	80 (52.63)	72 (47.37)	
	1	20	11 (55.00)	9 (45.00)	
	NA	2	0	2 (100.00)	

NA, No Available.

The bold values in indicate *p* < 0.05.

**Table 2:** Relationship between EFTUD2 expression and LUAD-related biomarkers.

Biomarker	Group	Total	EFTUD2 expression		*P*
			Low	High	
All patients		174	91 (52.30)	83 (47.70)	
CA125 (U/mL)					0.3507
	< 12.44	86	46 (53.48)	40 (46.51)	
	≥ 12.44	87	44 (50.57)	43 (49.43)	
	NA	1	1 (100.00)	0	
CEA (ng/mL)					0.0974
	< 3.26	86	49 (56.98)	37 (43.02)	
	≥ 3.26	87	41 (47.67)	46 (52.32)	
	NA	1	1 (100.00)	0	
**CYFRA-21-1** (ng/mL)					**0.0319**
	< 2.71	83	49 (59.03)	34 (40.96)	
	≥ 2.71	87	39 (44.83)	48 (55.17)	
	NA	4	3 (75.00)	1 (25.00)	
Pro-GRP (pg/mL)					0.3135
	< 46.58	81	44 (54.32)	37 (45.68)	
	≥ 46.58	87	44 (50.57)	43 (49.43)	
	NA	6	3 (50.00)	3 (50.00)	

CA125, carbohydrate antigen 125; CEA, carcinoembryonic antigen; CYFRA-21-1, cytokeratin-19 fragment antigen 21-1; Pro-GRP, pro-gastrin releasing peptide precursor.

**Table 3:** Relationship between EFTUD2 expression and clinical characteristics of patients with LUAD in TCGA.

Characteristic	Group	Total	EFTUD2 expression		*P*
			Low (%)	High (%)	
All patients		501	200	301	
**Age (years)**					**0.0276**
	≤ 65	219	77 (35.16)	142 (64.84)	
	> 65	282	123 (43.62)	159 (56.38)	
Sex					0.4843
	Male	231	92 (39.82)	139 (60.17)	
	Female	270	108 (40.00)	162 (60.00)	
**Smoking history**					**0.0135**
	Never	28	10 (35.71)	18 (64.29)	
	Reform	127	70 (55.12)	57 (44.88)	
	Current	46	15 (32.61)	31 (67.39)	
Pathologic stage					0.1756
	I	268	117 (43.66)	151 (56.34)	
	II	119	43 (36.13)	76 (63.87)	
	III	81	26 (32.10)	55 (67.90)	
	IV	25	8 (32.00)	17 (68.00)	
T stage					0.9868
	1	167	66 (39.52)	101 (60.48)	
	2	267	107 (40.07)	160 (59.93)	
	3	45	19 (42.22)	26 (57.78)	
	4	19	8 (42.11)	11 (57.89)	
**N stage**					**0.0364**
	0	324	137 (42.28)	187 (57.71)	
	1	94	38 (40.43)	56 (59.57)	
	2	72	20 (27.78)	52 (72.22)	
M stage					0.2432
	0	333	135 (40.54)	198 (59.46)	
	1	24	8 (33.33)	16 (66.67)	

The bold values in indicate *p* < 0.05.

**Table 4:** Univariate and multivariate Cox regression analysis of clinical characteristics.

Clinical characteristics	Uni-variables cox			Multi-variables cox		
	HR	95%CI	*P*	HR	95%CI	*P*
Age (years,mean ± SD)	1.009	0.994-1.024	2.64E-01	–	–	–
Gender (Male/Female)	1.061	0.792-1.418	6.95E-01	–	–	–
**Pathologic_M** **(M1/M0)**	2.111	1.232-3.616	**5.36E-03**	0.922	0.337-2.522	8.74E-01
**Pathologic_N** **(N2/N1/N0)**	1.782	1.493-2.128	**3.99E-11**	1.287	0.995-1.664	5.48E-02
**Pathologic_T** **(T4/T3/T2/T1)**	1.551	1.289-1.863	**3.10E-06**	1.236	1.0138 -1.506	**3.61E-02**
**Pathologic_stage** **(IV/III/II/I)**	1.679	1.463-1.928	**2.94E-14**	1.353	1.076-1.702	9.65E-03
Smoking history (Current/Reform/Never)	0.765	0.542-1.081	1.29E-01	–	–	–
**ETTUD2 level** **(High/Low)**	1.360	1.003-1.844	**4.67E-02**	1.145	0.834-1.573	4.03E-01

The bold values in indicate *p* < 0.05.

### 3.3 Expression of related hub genes indicates importance of EFTUD2 in LUAD diagnosis and prognosis

**Figure 2:**
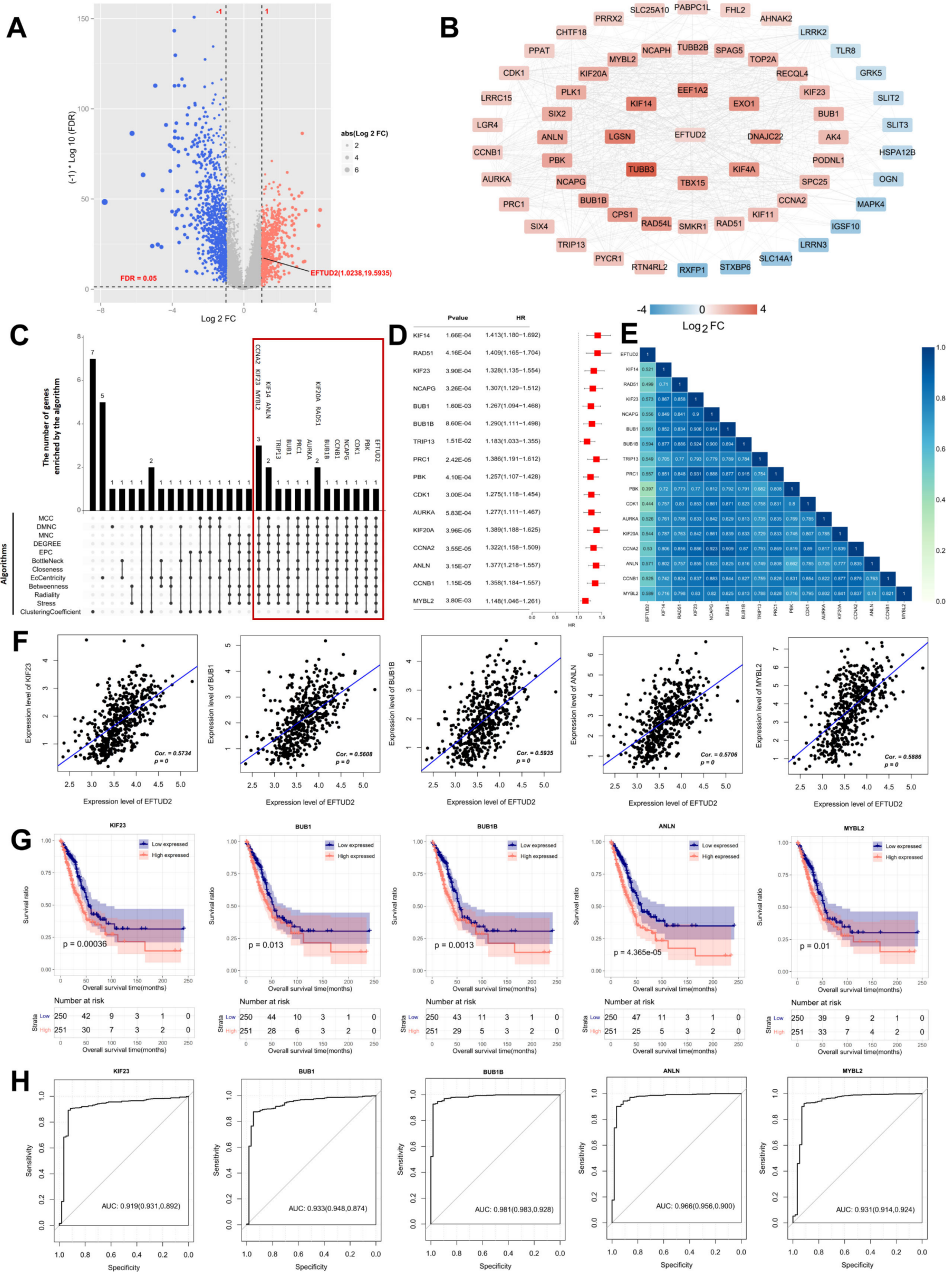
Screening of EFTUD2 coexpressed hub genes in LUAD, and evaluation of their clinical diagnostic and prognostic value. **(A)** Volcano plot of DEGs in LUAD versus normal lung tissues in TCGA dataset (p < 0.05, |log2FC| ≥ 1). **(B)** PPI network of EFTUD2-related DEGs in LUAD, with connection scores > 0.4. **(C)** Venn diagram of candidate hub genes screened by 12 algorithms. **(D)** Forest plot of univariate Cox regression analysis for the 16 EFTUD2-related hub genes identified in LUAD. **(E)** Heatmap of correlations between the 16 EFTUD2-related hub genes and EFTUD2. **(F)** Correlation analysis of significant EFTUD2-related hub genes. **(G)** Survival analysis of significant EFTUD2-related hub genes. **(H)** ROC curves of EFTUD2-related hub genes with AUC > 0.9.

To determine the molecular mechanism associated with EFTUD2, we screened DEGs between 58 normal donors and 501 patients with LUAD from the TCGA dataset. The volcano plot results included 344 downregulated and 354 upregulated DEGs; one of these upregulated DEGs was *EFTUD2* (**Figure 2A**). Then, the interactions between EFTUD2 and the screened DEGs were analyzed by using the STRING database; the results demonstrated 51 (red) and 13 (blue) proteins having strong positive and negative interactions with EFTUD2, respectively (**Figure 2B**). Hub genes related to EFTUD2 in the PPI network were further identified using 12 topology analysis algorithms, whereby the genes simultaneously identified by nine or more algorithms were retained as hub genes. In total, 16 hub genes were found to meet the screening criteria (**Figure 2C**). We next performed univariate Cox regression analysis on these 16 hub genes. The hazard ratios for all 16 hub genes were >1 (**Figure 2D**), indicating that they can be independent prognostic factors for LUAD. Moreover, EFTUD2 was significantly and positively correlated with all 16 hub genes (all *p* < 0.05; **Figure 2E**). The five genes most positively related to EFTUD2 were *KIF23*, *BUB1*, *BUB1B*, *ANLN*, and *MYBL2* (**Figure 2F**). CNV analysis was performed on 16 hub genes to investigate whether the expression levels of EFTUD2 and the hub genes are influenced by genetic mutations. The results indicated that, among the 16 hub genes, the expression of BUB1 (Spearman: 0.15) and TRIP13 (Spearman: 0.13) may be somewhat affected by copy number variations, while the correlation coefficients were comparatively weak (**Supplementary Figure S1E**). The survival analysis results demonstrated that strong expression of these five genes suggested poor OS in patients with LUAD (**Figure 2G**). The AUCs of all these five genes were >0.9 (**Figure 2H**), suggesting the significant diagnostic value of EFTUD2 in LUAD. The diagnostic and prognostic value of EFTUD2-related hub genes in LUAD indicated the crucial role of EFTUD2 in LUAD diagnosis and prognosis.

### 3.4 High EFTUD2 expression in LUAD affects the TIME

**Figure 3:**
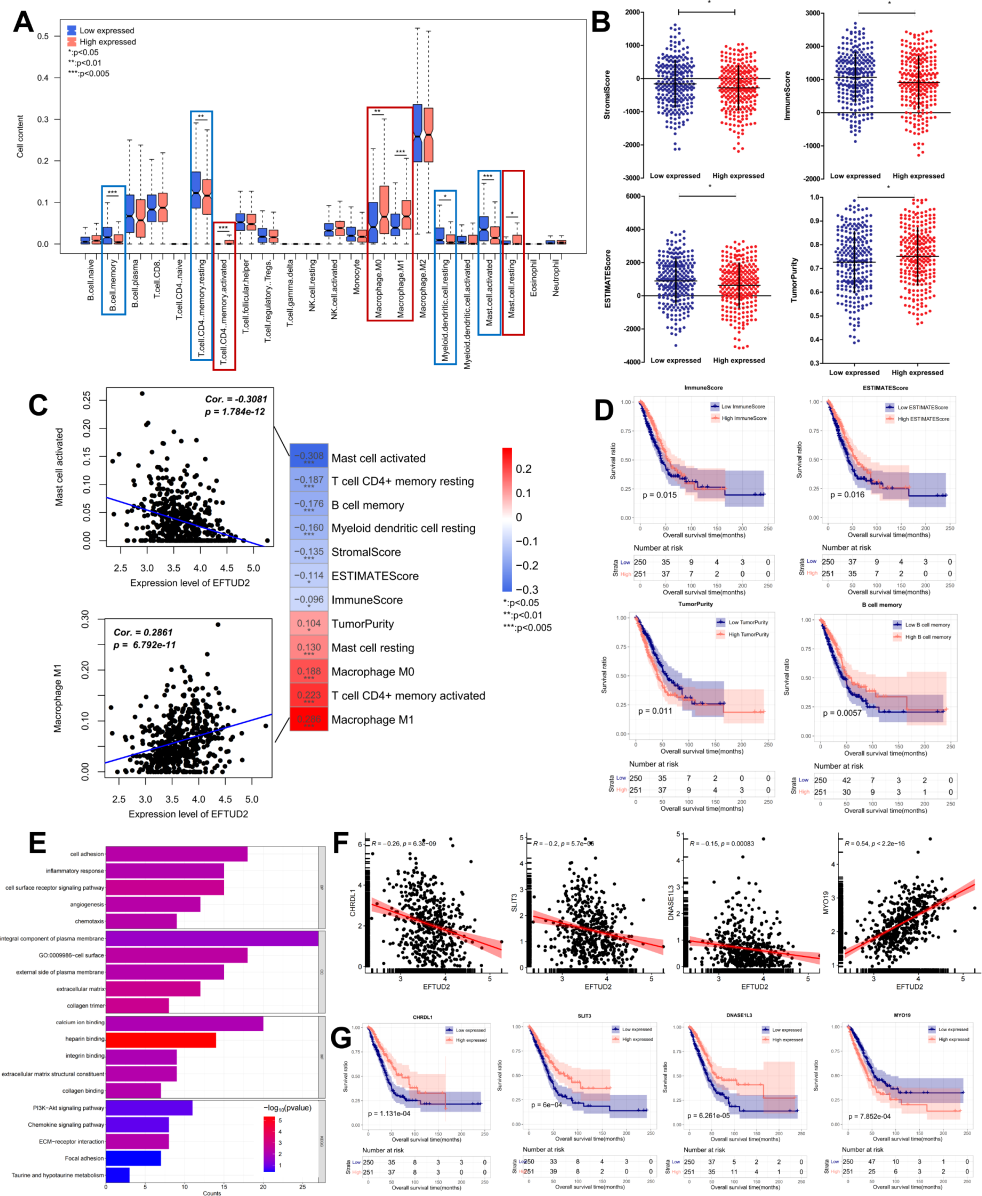
Impact of EFTUD2 on the TIME. **(A)** Immune cell distribution in LUAD tissues with high and low EFTUD2 expression. **(B)** ESTIMATE scores (TumorPurity, StromalScore, ImmuneScore, and ESTIMATEScore) in LUAD tissues with high and low EFTUD2 expression. **(C)** Correlation analysis of EFTUD2 with eight immune cell subtypes and ESTIMATE scores. **(D)** Survival analysis of memory B cells, ImmuneScore, ESTIMATEScore, and TumorPurity in LUAD. **(E)** GO and KEGG enrichment analyses of immune genes related to EFTUD2 (FDR < 0.05). **(F)** Correlation between EFTUD2 and related immune genes. **(G)** Survival analysis of EFTUD2-related immune genes in LUAD. *p < 0.05, **p < 0.01, ***p < 0.001.

Considering the close relationship of EFTUD2 with the immune system (17, 42), we assessed the association of EFTUD2 with LUAD TIME. First, we analyzed the association between EFTUD2 and immune cell subtypes based on LUAD expression profiles in the TCGA. Compared with patients with low EFTUD2 expression, patients with high EFTUD2 expression demonstrated significantly lower abundance of memory B cells, resting memory CD4+T cells, resting myeloid dendritic cells, and activated mast cells but significantly higher abundance of activated memory CD4+T cells, M0 macrophages, M1 macrophages, and resting mast cells (**Figure 3A**). This suggests that EFTUD2 may regulate the infiltration levels of memory B cells, T cells, macrophages, and mast cells in LUAD tumors. However, EFTUD2 expression was noted to be correlated negatively with activated mast cell, resting memory CD4+T cell, memory B cell, and resting myeloid dendritic cell abundance but positively with resting mast cell, macrophage M0, activated memory CD4+ T cell, and M1 macrophage abundance (**Figure 3C**). In terms of the TIME scores, EFTUD2 was correlated significantly and positively with TumorPurity but negatively with StromalScore, ImmuneScore, and ESTIMATEScore (**Figure 3C**). However, StromalScore, ImmuneScore, and ESTIMATEScore values were significantly lower but TumorPurity scores were higher in patients with high EFTUD2 expression than in patients with low EFTUD2 expression (**Figure 3B**). Survival analysis revealed that LUAD patients with low ESTIMATEScore and ImmuneScore values or high TumorPurity scores had shorter OS (**Figure 3D**). Moreover, patients with low memory B cell abundance had a worse prognosis (**Figure 3D**). High expression of EFTUD2 reduces the abundance of memory B cells in LUAD tissues, suggesting that EFTUD2 may lower patient prognosis by affecting the infiltration levels of memory B cells. The GO enrichment analysis results demonstrated that EFTUD2 expression was strongly associated with immune pathways, including those related to cell adhesion, chemotaxis, immune response, and cell surface receptor signaling. KEGG enrichment analysis results showed that EFTUD2 was strongly associated with the PI3K-Akt signaling, chemokine signaling, extracellular matrix receptor binding, and focal adhesion pathways (**Figure 3E**). The five immune-related genes most strongly associated with EFTUD2 are depicted in **Figure 3E**; of them, *CHRDL1*, *SLIT3*, and *DNASE1L3* were negatively correlated with EFTUD2, whereas *MTO19* was positively correlated with EFTUD2 (**Figure 3F**). Survival analysis revealed that patients with lower *CHRDL1*, *SLIT3*, and *DNASE1L3* expression or higher *MTO19* expression had significantly poorer prognosis (**Figure 3G**). These data suggest that EFTUD2 upregulation is strongly associated with an immune suppressive TIME.

### 3.5 EFTUD2 is strongly associated with the cGAS-STING pathway and m6A modification

**Figure 4:**
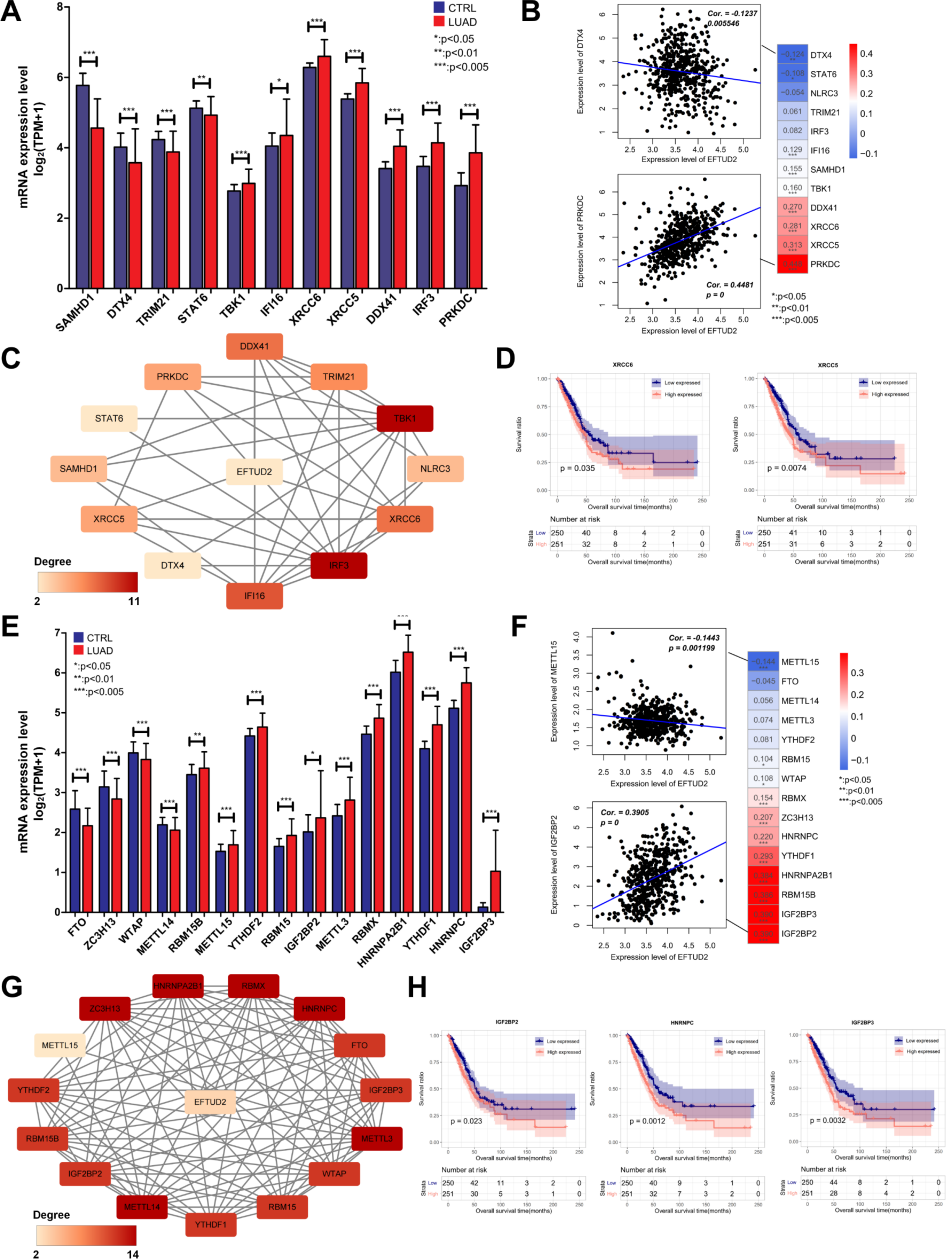
Correlation analysis of EFTUD2 with the cGAS-STING pathway and m6A modification in LUAD. **(A)** Expression of cGAS-STING pathway–related genes in normal and LUAD tissues. **(B)** Correlation of EFTUD2 with LUAD-specific cGAS-STING pathway genes. **(C)** PPI network of EFTUD2 and LUAD-specific cGAS-STING pathway genes. **(D)** Survival analysis for the cGAS-STING pathway genes XRCC6 and XRCC5. **(E)** Expression of m6A modification–related genes in normal and LUAD tissues. **(F)** Correlation of EFTUD2 with LUAD-specific m6A modification genes. **(G)** PPI network of EFTUD2 and LUAD-specific m6A modification genes. **(H)** Survival analysis of the m6A modification genes IGF2BP2, IGF2BP3, and HNRNPC. *p < 0.05, **p < 0.01, ***p < 0.001.

EFTUD2 is involved in innate immune responses (43). Given the complex relationship between the cGAS-STING pathway and innate immunity, we explored whether EFTUD2 is associated with the cGAS-STING pathway. We first analyzed the expression of m6A-related genes between normal and LUAD tissues and found that *SAMHD1*, *DTX4*, *TRIM21*, and *STAT6* expression was significantly lower and *TBK1*, *IFI16*, *XRCC6*, *XRCC5*, *DDX41*, *IRF3*, and *PRKDC* expression significantly higher in LUAD tissues (**Figure 4A**). Correlation analysis revealed that EFTUD2 was correlated positively with *IFI16*, *SAMHD1*, *TBK1*, *DDX41*, *XRCC6*, *XRCC5*, and *PRKDC* expression and negatively with *STAT6* and *DTX4* expression (**Figure 4B**). In addition, based on CNV analysis, we found that the expression levels of EFTUD2 were weakly positively correlated with the copy number variations of *TBK1*(Spearman: 0.15) and *SAMHD1*(Spearman: 0.18), whereas other genes did not show the same trend (**Supplementary Figure S1F**). This suggests that the elevated expression of *TBK1* and *SAMHD1* observed when EFTUD2 is upregulated may be partially influenced by copy number variations. The PPI demonstrated the presence of significant interactions between EFTUD2 and the aforementioned genes (**Figure 4C**). Moreover, our K–M survival analysis on these EFTUD2-related genes revealed that patients with high *XRCC6* and *XRCC5* expression had poor prognoses (**Figure 4D**). Thus, EFTUD2 may be involved in cGAS-STING pathway regulation.

Because m6A modification orchestrates mRNA precursor processing, EFTUD2 is involved in spliceosome activation (8). Thus, we explored associations between EFTUD2 and m6A modification and compared the differential expression of m6A-related genes in normal and LUAD tissues. The results demonstrated that *METTL14*, *RBM15B*, *METTL15*, *YTHDF2*, *RBM15*, *IGF2BP2*, *METTL3*, *RBMX*, *HNRNPA2B1*, *YTHDF1*, *HNRNPC*, and *IGF2BP3* were significantly overexpressed in LUAD tissues, whereas *FTO*, *ZC3H13*, and *WTAP* were significantly underexpressed (**Figure 4E**). We then analyzed the correlations between EFTUD2 and the aforementioned genes in patients with LUAD, and the results demonstrated that EFTUD2 expression was correlated positively with *RBM15*, *WTAP*, *RBMX*, *ZC3H13*, *HNRNPC*, *YTHDF1*, *HNRNPA2B1*, *RBM15B*, *IGF2BP3*, and *IGF2BP2* expression and negatively with *METTL15* expression (**Figure 4F**). Similarly, to assess whether changes in the expression levels of m6A genes were influenced by genetic mutations, we conducted CNV analysis. The results indicated that, among the m6A genes potentially affected by EFTUD2 levels, the mRNA levels of *IGFBP2* (Spearman: 0.19), *WTAP* (Spearman: 0.12), and *METTL15* (Spearman: -0.10) may be influenced by copy number variations to some extent; however, the correlation coefficients were relatively low (**Supplementary Figure S1G**). We also analyzed the interactions between EFTUD2 and the aforementioned m6A-associated genes and noted a robust correlation of EFTUD2 with the genes (**Figure 4G**). The K–M survival analysis revealed that *IGF2BP2*, *HNRNPC*, and *IGF2BP3* expression—positively correlated with EFTUD2—were associated with poor prognosis in patients with LUAD (**Figure 4H**). These results indicated that m6A modification is strongly associated with EFTUD2.

### 3.6 *EFTUD2* knockdown affects LUAD cell behavior

**Figure 5:**
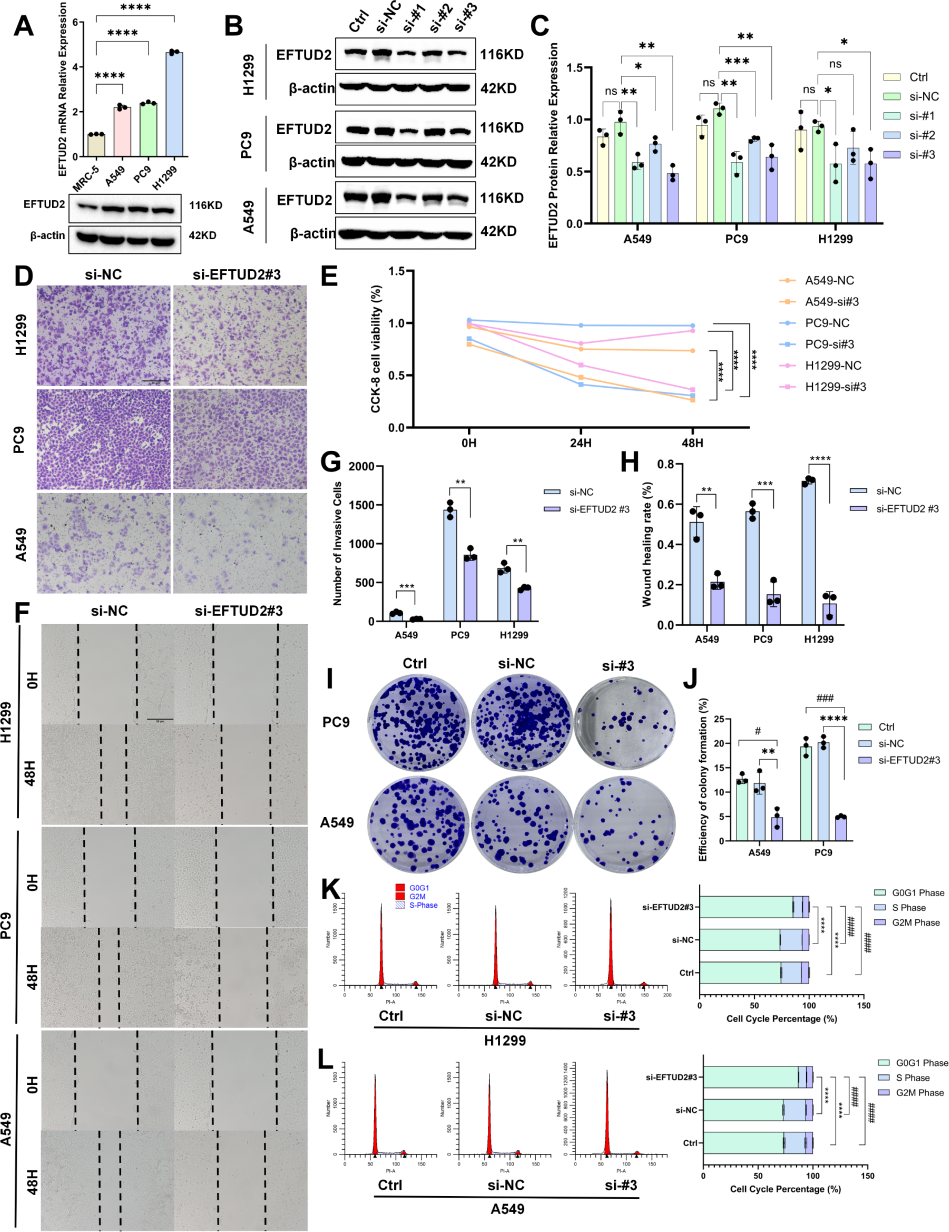
Impact of EFTUD2 knockdown on the proliferation, migration, invasion, tumorigenicity, and cell cycle progression of LUAD cells. **(A)** mRNA and protein expression of EFTUD2 in MRC-5, A549, PC9, and H1299 cells. **(B)** EFTUD2 protein expression in A549, PC9, and H1299 cells transfected with siRNA targeting EFTUD2 or negative control siRNA. **(C)** The quantitative results of EFTUD2 protein expression in A549, PC9, and H1299 cells transfected with three different siRNAs targeting EFTUD2, presented as means ± SDs. **(D)** A549, PC9, and H1299 cell invasion 48 h after transfection. **(E)** Cell proliferation of A549, PC9, and H1299 0, 24, and 48 h after transfection. **(F)** A549 and PC9 cell wound healing after EFTUD2 knockdown. **(G)** Numbers of invasive cells, presented as means ± SDs. **(H)** Numbers of migrating cells in wound healing assays, presented as means ± SDs. **(I)** Plate colony formation of A549 and PC9 cells transfected with siRNA targeting EFTUD2 or negative control siRNA. **(J)** Efficiency of colony formation (%), presented as means ± SDs. **(K, L)** Cell cycle in H1299 and A549 cells after EFTUD2 knockdown; * denotes the S phase, and # denotes the G0/G1 phase. *p < 0.05, **p < 0.01, ***p < 0.001, ****p < 0.0001, #*p* < 0.05, ###*p* < 0.001, ####*p* < 0.0001, and ns, not significant (*p* > 0.05).

To elucidate the biological functions of EFTUD2 in LUAD, we first detected *EFTUD2* mRNA and protein expression in the LUAD cell lines A549, H1299, and PC9 and the normal lung fibroblasts cell line MRC-5. The results demonstrated that *EFTUD2* expression was significantly higher in the LUAD cells than in the normal cells (**Figure 5A**). We knocked down *EFTUD2* expression in the LUAD cells by using three siRNAs targeting distinct sequences and then detected *EFTUD2* protein levels (**Figure 5B**). The results demonstrated that both si-EFTUD2#1 and si-EFTUD2#3 significantly interfered with *EFTUD2* expression. Compared to si-EFTUD2#1, si-EFTUD2#3 achieved the most effective knockdown in A549 cells while exhibiting relatively consistent knockdown efficiency in PC9 and H1299 cells (**Figure 5C**). Therefore, we selected si-EFTUD2#3 for subsequent experiments. CCK-8 assays revealed that the proliferation of the three LUAD cell lines significantly decreased after *EFTUD2* knockdown (**Figure 5E**). The transwell assay results demonstrated that in all three cell lines, compared with the negative control, si-EFTUD2#3 reduced cell invasion considerably (**Figures 5 D, G**). The wound healing assay results demonstrated that *EFTUD2* knockdown significantly attenuated wound healing in LUAD cells compared with the control group. (**Figures 5F, H**). Moreover, PC9 and A549 cells transfected with si-EFTUD2#3 demonstrated significant decreases in their colony formation ability, indicating that *EFTUD2* downregulation significantly weakened the tumorigenicity of LUAD cells (**Figures 5I, J**). Flow cytometry assays revealed that *EFTUD2* knockdown led to cell-cycle arrest at the G0/G1 phase in H1299 cells (**Figure 5K**) and A549 cells (**Figure 5L**). Thus, *EFTUD2* knockdown can inhibit proliferation, migration, invasion, tumorigenicity, and cell-cycle progression in LUAD cells.

### 3.7 *EFTUD2* upregulation correlates positively with tumor-associated pathway and glycolysis signatures

**Figure 6:**
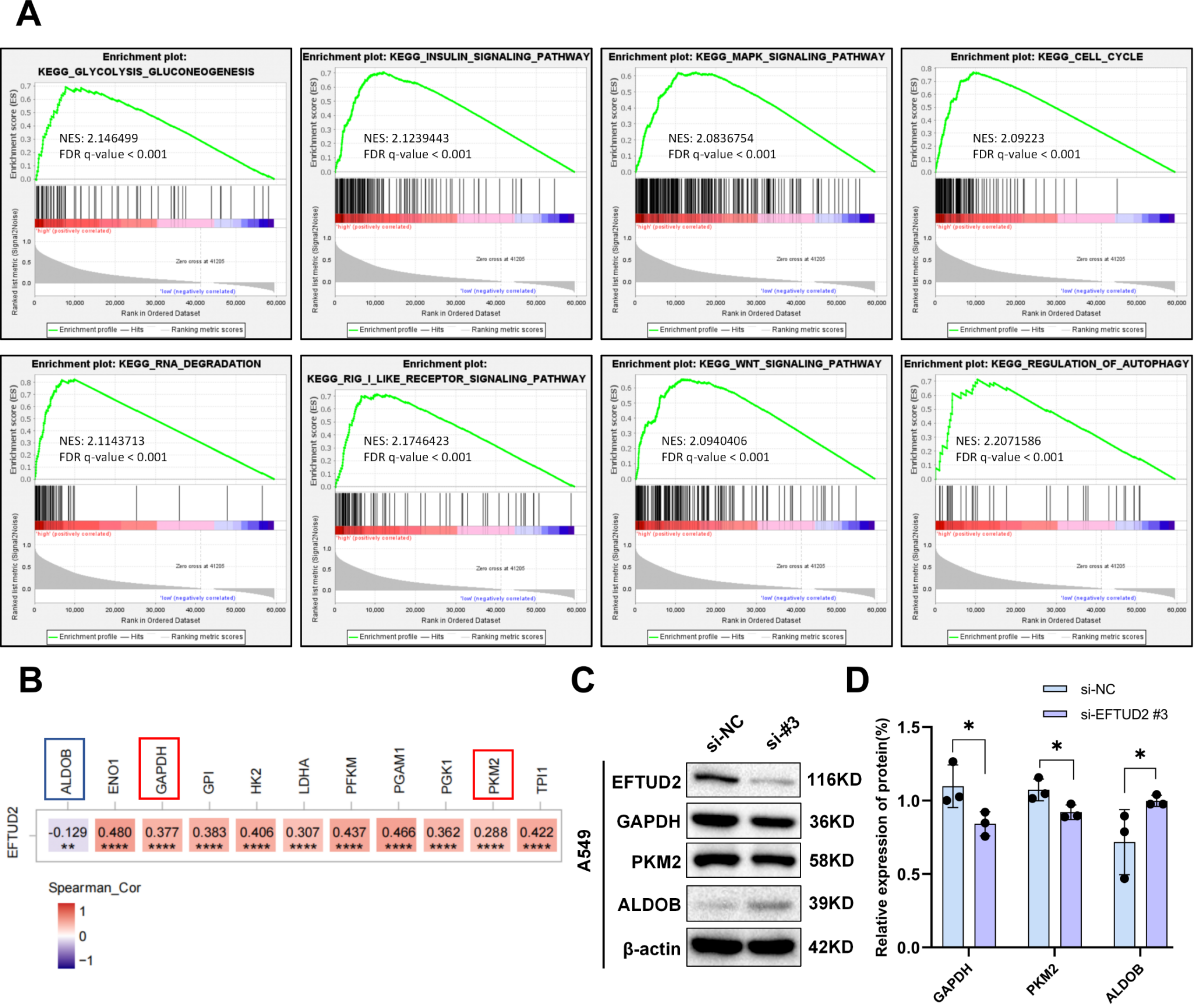
EFTUD2 involvement in multiple tumor-associated pathways in LUAD development and its promotion of tumor aerobic glycolysis. **(A)** GSEA based on EFTUD2 levels in LUAD. **(B)** Heatmap of correlation between EFTUD2 and key glycolytic enzymes in LUAD based on TCGA data from TIMER. **(C)** GAPDH, PKM2, and ALDOB protein expression in A549 cells after EFTUD2 knockdown. **(D)** GAPDH, PKM2, and ALDOB protein levels, presented as means ± SDs, in A549 cells after EFTUD2 knockdown. *p < 0.05, **p < 0.01, ****p < 0.0001.

To investigate the possible molecular mechanisms of *EFTUD2* in LUAD, we performed GSEA on genes in the low and high EFTUD2 expression groups of patients with LUAD from TCGA. The results demonstrated that *EFTUD2* upregulation is correlated with a prevalent enrichment of gene signatures across several pathways: the cell cycle, glycolysis, insulin signaling, MAPK, RIG-I-like receptor, wnt signaling, RNA degradation, and autophagy regulation (**Figure 6A**). Because glycolysis is a crucial metabolic process, providing energy to cancer cells, we examined the correlation between EFTUD2 and key enzymes involved in glycolysis in LUAD by using TIMER and identified significant associations between EFTUD2 and various regulatory enzymes including ENO1, glyceraldehyde 3-phosphate dehydrogenase (GAPDH), GPI, HK2, LDHA, PFKM, pyruvate kinase M2 (PKM2), PGAM1, PGK1, TPI1, and aldolase B (ALDOB; **Figure 6B**). We further investigated whether EFTUD2 is involved in glycolysis in the LUAD cell line A549. The results demonstrated that *EFTUD2* knockdown led to a significant decrease in PKM2 and GAPDH protein expression but a significant increase in ALDOB protein expression (**Figures 6C, D**).

### 3.8 EFTUD2 promotes glycolysis and lactate production in LUAD tumor xenografts

**Figure 7:**
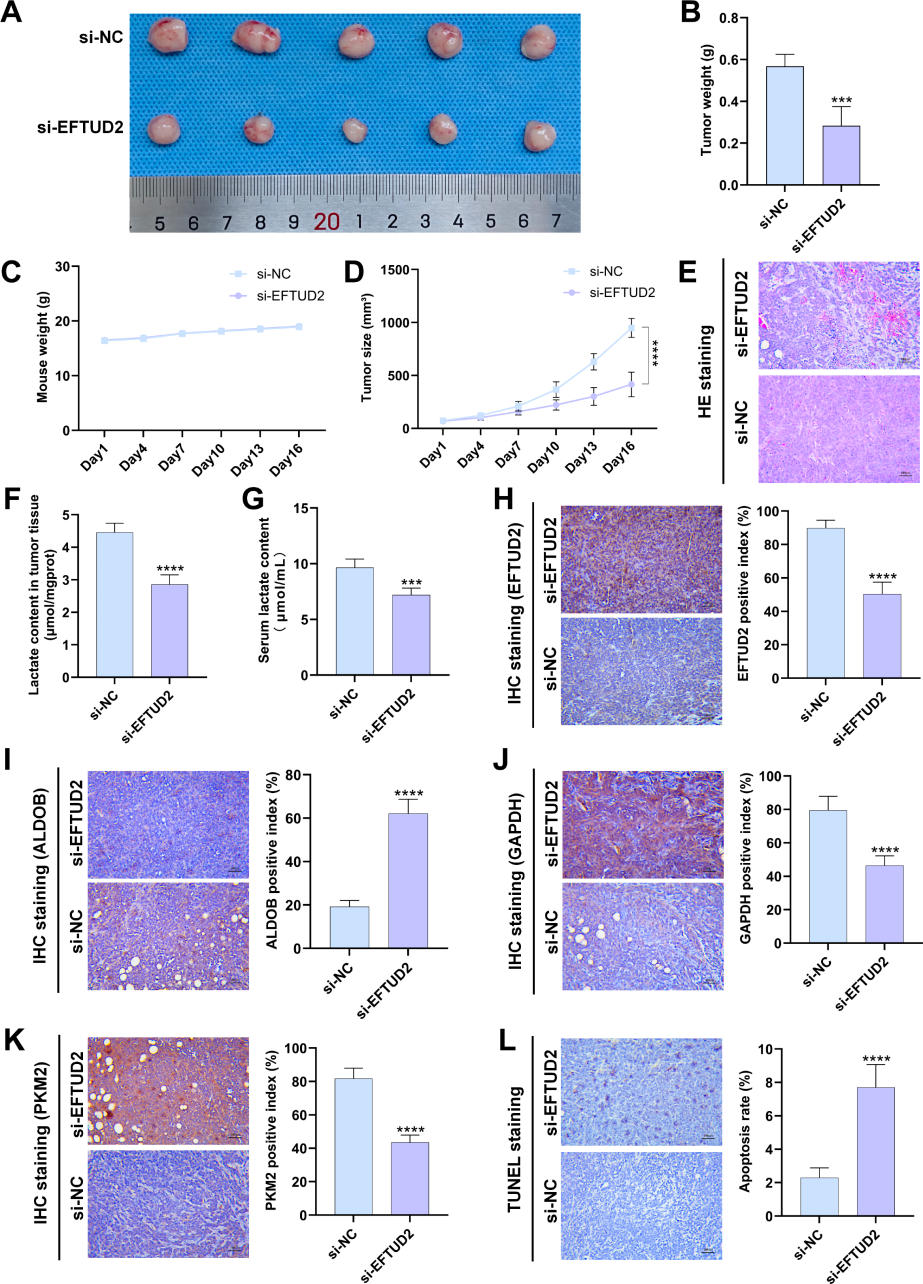
Effects of EFTUD2 knockdown on LUAD tumor growth, glycolysis, and apoptosis *in vivo*. **(A)** Photos of LUAD xenografts in the control and EFTUD2 knockdown groups. **(B)** Tumor weight of LUAD xenografts after EFTUD2 knockdown. **(C)** The tumor volume change curves of LUAD xenografts in the control and EFTUD2 knockdown groups. **(D)** The body weight change curves of mice bearing LUAD xenografts after EFTUD2 knockdown. **(E)** HE staining images of tumors after EFTUD2 knockdown. **(F)** Lactate content in tumor tissues after EFTUD2 knockdown. **(G)** Lactate content in the serum of mice after EFTUD2 knockdown before euthanasia. **(H-K)** IHC staining results of EFTUD2 **(H)**, ALDOB **(I)**, GAPDH **(J)**, and PKM2 **(K)** in tumor tissues after EFTUD2 knockdown. **(L)** Cell apoptosis level in LUAD tumor xenograft tissues after EFTUD2 knockdown. Data represent the mean ± SDs. n = 5 per group. ***p < 0.001, ****p < 0.0001.

We established a tumor xenograft model to investigate the role of EFTUD2 in mediating LUAD tumor growth *in vivo*. There was no significant difference in body weight between the control and knockdown groups throughout the mice’s growth period (**Figure 7C**). Over time, the growth of tumor volume in the EFTUD2 knockdown group gradually slowed down (**Figure 7D**). By day 16, the tumor volume and weight in the knockdown group were significantly lower than those in the control group (**Figures 7 A, B**). HE staining of the tumor xenografts revealed abundant microvessels and active tumor cell proliferation in the control group. In contrast, the EFTUD2 knockdown group displayed tumor necrosis foci, a reduced number of microvessels, and diminished tumor cell proliferation (**Figure 7E**).

We measured lactate levels in the serum and tumor tissues of the collected mouse samples. The results revealed that, compared to the control group, lactate levels in both serum and tumor tissues of the EFTUD2 knockdown mice were significantly reduced (**Figures 7F, G**). We further evaluated the effect of EFTUD2 on key glycolytic enzymes using IHC staining. Following EFTUD2 knockdown, the protein expression of the glycolytic enzymes GAPDH and PKM2 decreased, while the expression of the negative regulator ALDOB increased (**Figures 7H–K**). These findings are consistent with the results from the cell experiments shown in **Figure 6C**. Additionally, TUNEL assays on tumor samples revealed that, compared to tumors from control mice, the tumor regions in the EFTUD2 knockdown group exhibited increased apoptosis (**Figure 7L**). These data suggest that knocking down EFTUD2 inhibits LUAD tumor growth in mice and suppresses glycolytic activity. Conversely, overactivation of EFTUD2 may promote LUAD tumor proliferation and elevate lactate metabolism levels in both the tumors and the host.

## 4 Discussion

In the present study, we noted that EFTUD2 was strongly expressed in LUAD tissues by using data from online databases and confirmed these results in patient LUAD tissue samples and cell lines—consistent with its expression patterns in colorectal (24), hepatocellular (22), and endometrial (23) cancers. This suggests that EFTUD2 may be an important potential risk factor in cancer development. Analysis of bioinformatics data and clinical characteristics both revealed that elevated EFTUD2 expression in LUAD is correlated with age and N classification (i.e., local lymph node metastasis). Clinical characteristic analysis revealed that elevated EFTUD2 may contribute to pleural invasion and intravascular tumor thrombosis in LUAD patients. In addition, the bioinformatics analysis results demonstrated that strong EFTUD2 expression is related to smoking history. The discrepancy between bioinformatics and clinical data analyses might be due to an insufficient clinical specimen sample size. High EFTUD2 expression was also closely related to CYFRA-21-1, which has a significant diagnostic and prognostic value in LUAD (41, 44). Consistent with those for HCC (20) and endometrial cancer (23), our results also demonstrated that EFTUD2 not only functions as a poor prognosis indicator but also has a substantial diagnostic value in LUAD. *In vivo* tumor xenograft experiments showed that after EFTUD2 knockdown, the TUNEL-positive cell rate increased, indicating enhanced cell apoptosis. Moreover, reducing elevated EFTUD2 expression in LUAD cells led to considerable reductions in LUAD cell proliferation and tumorigenicity. Thus, high EFTUD2 expression is a potential biomarker of LUAD and may play an auxiliary role in its diagnosis and targeted therapy. Building on this, future research should focus on validating EFTUD2 in larger patient cohorts to confirm its clinical relevance and prognostic value.

Among EFTUD2-related hub genes, *BUB1* and *BUB1B* are spindle assembly checkpoint genes that play pivotal roles in cell-cycle regulation (45), whereas *KIF23* encodes mitotic kinesin-like protein 1, essential for cytokinesis (46). Thus, EFTUD2 may be closely associated with the cell cycle; this was confirmed through flow cytometry: *EFTUD2* knockdown led LUAD cells to become arrested in the G0/G1 phase of the cell cycle, suggesting that high EFTUD2 expression promotes cell-cycle progression in LUAD. The GSEA analysis results also showed that EFTUD2 positively regulates the cell cycle pathway, providing strong evidence for its role in the cell cycle. Moreover, BUB1 is a potential biomarker for predicting outcomes in liver intrahepatic cholangiocarcinoma and gauging the condition’s immune profile (47). Increased BUB1B expression, achieved through m6A modification, can restore the malignant characteristics, self-renewal capabilities, and resistance to immune responses in cancer stem cells (48). Similarly, ANLN exhibits increased expression across various cancers and thus is considered an oncoimmunological biomarker (49). MYBL2 is overexpressed in various cancers, and it regulates proliferation, progression, and immune infiltration in all cancers (50–52). Elevated KIF23 expression has been linked to poorer cancer outcomes, and it is correlated with immune cell permeation and reaction to immunotherapeutic treatments (53, 54). The close relationship of these EFTUD2-associated genes with immune infiltration and responses in cancer cells suggests that in LUAD, strong EFTUD2 expression may have a complex relationship with immune responses.

Through CIBERSORT analysis, we noted that altered EFTUD2 expression affects the recruitment of various immune cells, such as memory B cells, memory CD4+T cells, resting myeloid dendritic cells, mast cells, and macrophages. Thus, EFTUD2 may influence both innate and adaptive immunity. Liu H et al. also explored the tumor microenvironment by studying immune cell infiltration. They validated the impact of breast cancer-associated genes on the TIME through CD8+ T cells (55). Among patients with decreased memory B cell infiltration, the prognosis is poorer. EFTUD2 expression is negatively correlated with the infiltration level of memory B cells. This suggests that when EFTUD2 is abnormally highly expressed, memory B cell infiltration is reduced, which may negatively impact the prognosis of LUAD patients. Notably, patients with high EFTUD2 expression demonstrated a decrease in activated mast cell infiltration but a significant increase in quiescent mast cell infiltration. Activated mast cells are among the first immune cell types recruited to the vicinity of tumors, capable of releasing various active chemokines and cytokines, further promoting immune cell infiltration (56, 57). Our findings suggested that elevated EFTUD2 expression suppresses mast cell activation or infiltration, subsequently affecting the infiltration of other immune cells.

The stromal and immune scores were employed to assess the degrees of stromal and immune cell infiltration, respectively—which provided a foundation for the ESTIMATE score to determine tumor purity within tumor tissues (58). In our ESTIMATE analysis, the high EFTUD2 expression group demonstrated lower StromalScore, ImmuneScore, and ESTIMATEScore values but higher TumorPurity scores; in other words, higher EFTUD2 expression may lead to the formation of a microenvironment with fewer immune and stromal cells and more tumor cells, which is consistent with the result that stromal and immune cell mixtures are negatively correlated TumorPurity scores (58–60). Immune score, a quantitative metric based on two lymphocyte populations, has been proposed to be a major prognostic factor in patients with cancer (61), whereas stromal score is also a valuable predictor of outcomes in patients with cancer (62). Here, we found that low StromalScore, ImmuneScore, and ESTIMATEScore values and high TumorPurity scores were associated with poor LUAD prognosis, indicating that EFTUD2 overexpression may shape a microenvironment detrimental to LUAD prognosis through alterations in immune, stromal, and tumor cell abundance.

The GO and KEGG enrichment analysis of EFTUD2-related immune genes demonstrated that these genes were mainly associated with cell adhesion, chemotaxis, and cell surface receptor binding. The chemotaxis of chemokines and cell adhesion molecules regulates various cancer development stages such as invasion and metastasis (63, 64). The binding of cell surface receptors to their ligands regulates intercellular communication and signal transduction between cancer and stromal cells, affecting cancer cell phenotypes in the tumor microenvironment (65). Therefore, we speculated that EFTUD2 controls LUAD cell invasion. Our *in vitro* invasion and wound-healing assays demonstrated that high EFTUD2 expression promoted LUAD cell invasion; however, the detailed underlying mechanisms warrant further analysis. Among the four immune genes most closely associated with EFTUD2, high expression levels of *CHRDL1*, *SLIT3*, and *DNASE1L3* are associated with better prognosis in LUAD patients. However, the expression patterns of these genes in LUAD are opposite to that of *EFTUD2*. This precisely indicates that when EFTUD2 is highly expressed, the reduced levels of CHRDL1, SLIT3, and DNASE1L3 can have a poorer prognosis for LUAD patients. In contrast, the expression patterns and prognostic value of *MYO19* are consistent with those of *EFTUD2* in LUAD. CHRDL1 and SLIT3 downregulation contributes to the invasive behavior and progression of colorectal cancer cells and thyroid malignancies (66, 67), and decreased DNASE1L3 expression may be a novel diagnostic and prognostic biomarker associated with immune infiltrates in LUAD (68); these results are consistent with our findings. In contrast to our results, Ren et al. discovered that high *MYO19* expression is negatively associated with tumor metastasis (69), which is a discrepancy potentially arising from MYO19 playing different roles in distinct types of cancer. Sheng M et al. recently reported that MYO19 is upregulated in non-small cell lung cancer (NSCLC) and can enhance cancer cell migration, promoting the expression of EMT markers (70). This is in strong agreement with our research findings. Thus, EFTUD2 overexpression may influence signaling transduction between cells and the matrix through interactions with these immune genes, thereby creating a microenvironment that facilitates LUAD cell migration and invasion.

In a study, time-course RNA-seq profiling of mouse embryonic fibroblasts revealed that EFTUD2 is associated with the cGAS-STING pathway (43). However, whether EFTUD2 is associated with the cGAS-STING pathway in LUAD remains unclear. Here, we screened nine cGAS-STING pathway–related DEGs between LUAD and normal tissues; of them, *XRCC6*, *XRCC5*, *PRKDC*, *DDX41*, *IFI16*, *SAMHD1*, and *TBK1* were correlated positively with EFTUD2, whereas *STAT6* and *DTX4* were correlated negatively with EFTUD2. Further correlation analysis revealed that in patients with LUAD, shorter OS with higher *XRCC6* and *XRCC5* expression, indicating that EFTUD2 is closely associated with the cGAS-STING pathway in LUAD. The XRCC5-XRCC6 dimer, along with PRKDC, forms a small subunit combining with the HDP-RNP complex to serve as an IRF3 activation platform (71, 72). SAMHD1 inhibits cGAS-STING pathway–mediated innate and adaptive immunity, and the absence of SAMHD1 reduces tumor-free survival (73). TBK1, a key fraction of the type I interferon signaling pathway, is activated by various DNA and RNA sensors, including IFI16 (74) and DDX41 (75). Cui et al. reported that NLRP4-DTX4 activates TBK1 for ubiquitination and degradation (76). Because DTX4 demonstrated a significant negative correlation with EFTUD2 expression, we hypothesized that EFTUD2 maintains TBK1 expression by inhibiting DTX4 expression; however, further investigation to confirm the underlying mechanisms is warranted. STAT6 expression triggers a suite of chemokines, including CCL20 (77) and CCL2 (78), which attract various immune cells such as B cells, T cells, macrophages, and dendritic cells and facilitate their infiltration. Thus, elevated EFTUD2 expression and its engagement with the aforementioned cGAS-STING pathway–related genes may foster an inflammatory and immunological context favoring LUAD progression.

m6A is a prevalent form of posttranscriptional RNA modification, and m6A dysregulation plays a role in lung tumor development and progression (79). The expression patterns of m6A regulators are correlated with the immune landscape in LUAD (80). Diao et al. have reported that EFTUD2 interacts with and mediates the ubiquitination of the m6A regulator YTHDF3 (81). Here, we noted that EFTUD2 mainly exhibited significant positive correlations with the m6a regulators RBM15, WTAP, RBMX, ZC3H13, HNRNPC, YTHDF1, HNRNPA2B1, RBM15B, IGF2BP3, and IGF2BP2. Moreover, LUAD patients with higher IGF2BP2, HNRNPC, and IGF2BP3 expression demonstrated poorer prognoses. As an m6A methylation reader, IGF2BP2 triggers endothelial cell activation, facilitating angiogenesis and metastatic spread of LUAD cells (82), and IGF2BP2 is considered a potential immune biomarker in head and neck squamous cell carcinoma (83). Enhanced HNRNPC expression is strongly associated with tumor stage advancement and metastasis development (84), and HNRNPC is considered a predictor for the response to immunotherapy in non-small cell lung cancer cells (85). *IGF2BP3*, an oncogene involved in LUAD (86, 87), mediates m6A modification, promoting epithelial–mesenchymal transition and LUAD metastasis (88). Blocking IGF2BP3 boosted antitumor immune responses by facilitating T-cell activation, countering exhaustion, and promoting infiltration via the programmed death ligand 1 pathway (89, 90). EFTUD2 is positively correlated with the expression of the above genes, suggesting that EFTUD2 may be involved in the positive regulation of these cellular activities in LUAD and contribute to a poorer prognosis for patients. Taken together, our findings, along with those reported previously, indicated that EFTUD2 overexpression may interact with m6A regulators to form a TIME that promotes LUAD cell metastasis.

Notably, both HNRNPC and IGF2BP2 promote tumor aerobic glycolysis (91, 92), and our GSEA revealed that EFTUD2 upregulation was significantly and positively correlated with glycolysis. Cancer cells have developed numerous strategies to boost glycolysis, forming the core of their metabolic processes. Oncogenic signals accelerate metabolic functions of glycolytic enzymes, primarily by increasing the enzyme expression or altering the enzymes after synthesis (93). In the current *in vitro* and *in vivo* study, *EFTUD2* knockdown led to a significant decrease in the expression of key glycolytic enzymes GAPDH and PKM2 but an increase in ALDOB expression, suggesting that EFTUD2 influences glycolysis in LUAD cells by regulating glycolytic enzyme expression. PKM2, a pyruvate kinase subtype, is a key enzyme limiting the glycolysis rate and significantly modulating tumor inflammatory metabolism (94). PKM2, a prognostic biomarker for LUAD, controls the TIME by regulating immune infiltration (95). *GAPDH*, typically recognized as a housekeeping gene, might play oncogenic roles across various cancers (96). Serotonylation of GAPDH prompts a metabolic reorientation toward glycolysis in CD8+T cells, bolstering their contribution to antitumor immunity (97). ALDOB facilitates a reversible metabolic process in which fructose-1,6-bisphosphate is transformed into dihydroxyacetone phosphate and glyceraldehyde-3-phosphate in glycolysis. Recently, ALDOB has been reported to be a tumor growth inhibitor (98, 99). A decrease in ALDOB expression is negatively associated with the presence of CD8+T cells in HCC tumor tissues, potentially allowing cancer cells to elude immune detection and affecting their susceptibility to immunotherapy (100). By monitoring the lactate levels in tumor tissues and serum in the later stages after tumor xenograft implantation in mice, we found that lactate levels in both serum and tumor tissues decreased following EFTUD2 knockdown. This is consistent with the aforementioned results, suggesting that the effect of EFTUD2 knockdown on glycolysis extends beyond key enzyme proteins and directly impacts lactate metabolism. Therefore, overexpressed EFTUD2 may participate in glycolysis by regulating PKM2, GAPDH, and ALDOB expression, thereby allowing LUAD cells to evade immune surveillance. In addition to the glycolysis pathway, GSEA analysis also showed that EFTUD2 is involved in regulating the insulin signaling pathway, which may be related to promoting glucose uptake by cells, thereby providing substrates for glycolysis.

Currently, tumor biomarkers explored from different perspectives of cancer using bioinformatic approaches encompass tumor diagnosis, prognosis, and therapy (101–104). Our study emphasizes the promoting role of elevated EFTUD2 expression in the progression of LUAD, and also includes its diagnostic and prognostic value for the tumor. The TCGA provides large-scale genomic data, but such large-scale data may also be affected by technical and biological biases (105). Further multi-omics studies are crucial for exploring the clinical value of EFTUD2 in LUAD (106). As an important splicing factor protein, EFTUD2 may indirectly promote the progression of LUAD by affecting the RNA splicing of other tumor-related genes. Similar mechanisms have been reported in tumor such as colitis-associated cancer (CAC) (24). Therefore, future studies should consider these alternative mechanisms to more comprehensively reveal the potential role of EFTUD2 in LUAD. Although our results suggest that EFTUD2 has significant value in the diagnosis and prognosis of LUAD, with the Cox univariate analysis showing a significant prognostic association (HR > 1), the multivariate analysis did not yield significant results. This indicates that the prognostic value of EFTUD2 in LUAD may be more closely related to disease progression. Furthermore, our study primarily focuses on the expression of EFTUD2 in LUAD tissues, but this approach may have limitations in both the study and clinical application of EFTUD2 in LUAD.

## 5 Conclusions

In this study, we first analyzed EFTUD2 expression and its relationship with the TIME, the cGAS-STING pathway, m6A modification, and glycolysis in LUAD. We noted that LUAD tissues demonstrate increased EFTUD2 expression, indicating its potential as a prognostic and diagnostic marker for LUAD. Moreover, high EFTUD2 expression was noted to be correlated with immune infiltration and influence the TIME to promote metastasis in LUAD. The interactions of EFTUD2 with genes related to the cGAS-STING pathway and m6A modification form an immunological context favoring LUAD progression and metastasis. Furthermore, EFTUD2 may promote the development of a tumor immune microenvironment (TIME) that facilitates immune escape in LUAD tumor cells by regulating the expression of glycolytic enzymes. In the future, additional *in vitro* and *in vivo* experiments clarifying the molecular mechanisms underlying EFTUD2 expression in relation to LUAD immunity and glycolysis further are warranted.

## Data Availability

The original contributions presented in the study are included in the article/**Supplementary Material**. Further inquiries can be directed to the corresponding authors.
