# Supplementary material for: EFTUD2 is a promising diagnostic and prognostic indicator involved in the tumor immune microenvironment and glycolysis of lung adenocarcinoma
Source: Front Oncol. 2025 Apr 1;15:1499217. doi: 10.3389/fonc.2025.1499217 (PMC11996642; doi:10.3389/fonc.2025.1499217)
Supplement: Supplementary file 1 [file DataSheet1.docx]

Supplementary Material

# Supplementary Tables

**Table S1.** Primers sequence of EFTUD2.

| **Primer** | **Sequence** |
| --- | --- |
| EFTUD2-F | CTTATTACAAGCTGCGCCACATT |
| EFTUD2-R | CGCGTCTTAGGGTTGAAGTAGAT |

**Table S2.** EFTUD2-targeting siRNA sequences.

| **No.** | **Gene** | **Sense (5'-3')** | **Antisense (5'-3')** |
| --- | --- | --- | --- |
| Si-1 | EFTUD2-  Homo-1066 | CACCUUUGGUGACAUUAAUTT | AUUAAUGUCACCAAAGGUGTT |
| Si-2 | EFTUD2-  Homo-437 | GCGGAUCUGAUGGAUAACUTT | AGUUAUCCAUCAGAUCCGCTT |
| Si-3 | EFTUD2-  Homo-886 | GCCUCCAACUGAUGCUUAUTT | AUAAGCAUCAGUUGGAGGCTT |

# Supplementary Figures


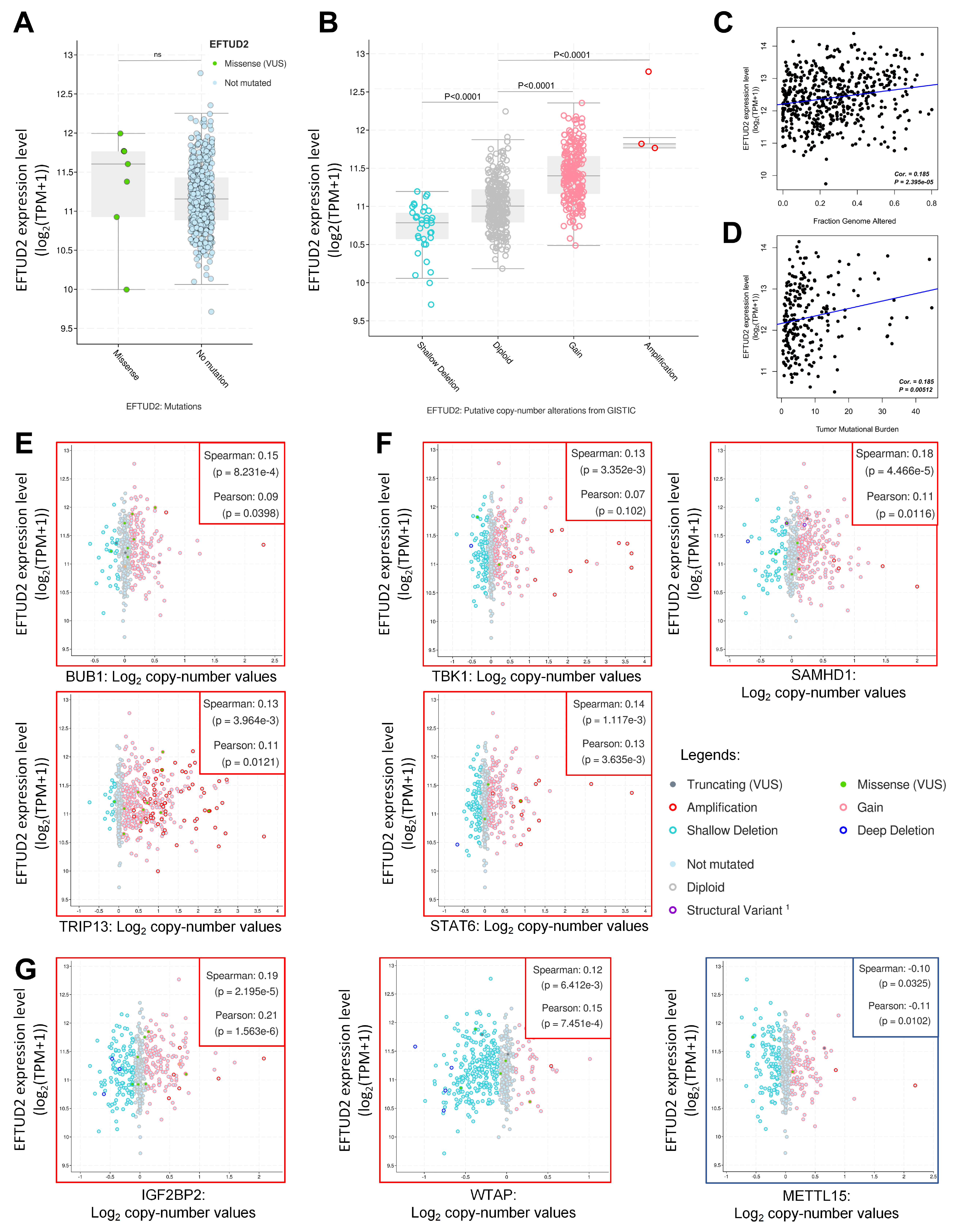


**Figure S1. The impact of variations in EFTUD2 on its expression levels, as well as the genomic mutation landscape of EFTUD2's differentially expressed genes (DEGs).** (A) The expression levels of EFTUD2 in LUAD patients with SNV mutations. (B) The expression levels of EFTUD2 in LUAD patients with CNV mutations. (C) The correlation between EFTUD2 expression and the fraction genome altered (FGA). (D) The correlation between EFTUD2 expression and the tumor mutational burden (TMB). (E) The correlation between the mutation degree of DEGs with the same gene mutation trend and the EFTUD2 level. (F) The correlation between the mutation degree of key genes that may be regulated by EFTUD2 and have the same trend as the cGAS - STING pathway and the EFTUD2 level. (G) The correlation between the mutation degree of key genes that may be regulated by EFTUD2 and have the same trend as the m6A pathway and the EFTUD2 level.
